# Potential biomarkers and signaling pathways associated with the pathogenesis of primary salivary gland carcinoma: a bioinformatics study

**DOI:** 10.5808/gi.21052

**Published:** 2021-12-31

**Authors:** Zeynab Bayat, Fatemeh Ahmadi-Motamayel, Mohadeseh Salimi Parsa, Amir Taherkhani

**Affiliations:** 1 Department of Oral and Maxillofacial Medicine, Faculty of Dentistry, Hamadan University of Medical Sciences, Hamadan 6517838678 Iran; 2 Dental Implants Research Center and Dental Research Center, Department of Oral Medicine, Hamadan University of Medical Sciences, Hamadan 6517838678, Iran; 3 Research Center for Molecular Medicine, Hamadan University of Medical Sciences, Hamadan 6517838678, Iran

**Keywords:** biomarkers, gene regulatory network, pathogenesis, protein-protein interaction network, salivary gland carcinoma

## Abstract

Salivary gland carcinoma (SGC) is rare cancer, constituting 6% of neoplasms in the head and neck area. The most responsible genes and pathways involved in the pathology of this disorder have not been fully understood. We aimed to identify differentially expressed genes (DEGs), the most critical hub genes, transcription factors, signaling pathways, and biological processes (BPs) associated with the pathogenesis of primary SGC. The mRNA dataset GSE153283 in the Gene Expression Omnibus database was re-analyzed for determining DEGs in cancer tissue of patients with primary SGC compared to the adjacent normal tissue (adjusted p-value < 0.001; |Log2 fold change| > 1). A protein interaction map (PIM) was built, and the main modules within the network were identified and focused on the different pathways and BP analyses. The hub genes of PIM were discovered, and their associated gene regulatory network was built to determine the master regulators involved in the pathogenesis of primary SGC. A total of 137 genes were found to be differentially expressed in primary SGC. The most significant pathways and BPs that were deregulated in the primary disease condition were associated with the cell cycle and fibroblast proliferation procedures. *TP53*, *EGF*, *FN1*, *NOTCH1*, *EZH2*, *COL1A1*, *SPP1*, *CDKN2A*, *WNT5A*, *PDGFRB*, *CCNB1*, and *H2AFX* were demonstrated to be the most critical genes linked with the primary SGC. *SPIB*, *FOXM1*, and *POLR2A* significantly regulate all the hub genes. This study illustrated several hub genes and their master regulators that might be appropriate targets for the therapeutic aims of primary SGC.

## Introduction

Salivary gland carcinoma (SGC) is an uncommon neoplasm, which constitutes approximately 6 percent of cancers in the head and neck region [1,2]. It is a heterogeneous disease, and therefore, the World Health Organization has classified the SGC into more than 30 histological subgroups [3]. Accordingly, mucoepidermoid carcinoma (MEC) is the most frequent subtype, with more than 30% of the SGC cases, followed by the adenoid cystic carcinoma (ACC) with the pathology in 23.8% of all SGC cases, and the adenocarcinoma not otherwise specified [4]. Despite many types of investigation that have been performed, the most critical genes and signaling pathways taking part in the etiology of the disease have not been fully demonstrated [5]. SGC can be treated at early stages by surgery, although patients with advanced stages of SGC present poor prognosis [6,7]. Previous studies have reported that chemotherapy and radiation therapy are low effective and have several side effects such as hyposalivation, neutropenia, swallowing disorder, and several neurological difficulties [8,9]. Recently, immunological approaches have been used for therapeutic aims in patients with advanced stages of the disease; however, many patients are resistant to immunotherapeutic strategies. Therefore, identifying new biomarkers responsible for the development and progression of SGC may be helpful in therapeutic approaches in future studies [6,10,11]. Re-analyzing high-throughput datasets (e.g., microarray datasets) has become an interesting strategy for researchers to achieve new and more significant facts associated with the pathogenesis of disorders such as neoplastic diseases, leading to the identification of novel therapeutic, prognostic, and diagnostic biomarkers [12-16].

Here, we hypothesized that significant alterations in the transcription of genes in the cancerous tissue of patients with SGC compared to the adjacent normal tissue might lead to abnormal activity of many biological procedures linked to the etiology of SGC. In addition, protein interaction map (PIM) and gene regulatory network (GRN) analysis may illustrate the hub genes and transcription factors (TFs) responsible for the occurrence and development of SGC [17]. Therefore, we re-analyzed the dataset GSE153283 to achieve differentially expressed genes (DEGs) (adjusted p-value [Padj] < 0.001; |Log2 fold change [FC]| > 1) between the cancer tissue of patients with primary SGC and the adjacent normal region. After that, a PIM and a GRN of the disease were constructed and analyzed. This study revealed the hub genes, the most significant clusters within the PIM, biological procedures responsible for primary SGC, and the common DEGs, hubs, and TFs among different subtypes of SGC.

The dataset GSE153283 was created by Meinrath et al. [5] to study the underlying mechanisms and signaling pathways associated with primary salivary gland carcinomas. The dataset consisted of 34 primary SGC (including ACC, MEC, and salivary duct carcinomas [SDC]) and 34 corresponding normal areas. Tissue samples were collected from patients at the University Hospital of Cologne, Germany, from 1990 to July 2014. The samples were then achieved from the tissue bank of the University Hospital of Cologne, Germany. All cancerous/healthy samples were collected from formalin-fixed and paraffin-embedded tissues with the aim of RNA derivation. The mRNA expression of 770 genes, primarily involved in well-known cancer-associated signaling pathways, was studied using GSE153283.

## Methods

### Statistical analysis

The dataset GSE153283 [5] within the Gene Expression Omnibus (GEO) (NCBI GEO, http://www.ncbi.nlm.nih.gov/geo) [18] was analyzed using the GEO2R tool [19]. The GSE153283 was constructed according to the platform of GPL19956 (NanoString nCounter human panCancer pathways Panel), including 34 PGC (including ACC, MEC, and SDC) and 34 adjacent healthy tissues. DEGs with characteristics of the corrected p-values less than 0.001 and |Log2 FC| more than one were considered statistically significant. The Benjamini & Hochberg analysis was adjusted to reach the corrected p-values.

### PIM and module analysis

The potential interactions between DEGs were detected by utilizing the Search Tool for the Retrieval of Interacting Genes (STRING) web-tool (http://string-db.org) [20]. The single nodes were then eliminated before importing the network into Cytoscape 3.8.2 (http://www.cytoscape.org) [21]. After that, the centrality of the genes was determined by the Network analyzer tool, and the most considerable connecting regions inside the PIM were discovered via the molecular complex detection (MCODE) plugin. The modules with characteristics of MCODE score > 3, k-score = 2, Max depth = 100, Degree cutoff = 2, and the minimum number of genes = 10 were selected for further functional analysis. MCODE is widely used to identify compressed parts of the graph named clusters, usually participating in common signaling pathways and/or biological processes (BPs). Furthermore, the MCODE identifies the seed node in each module, which is the main gene involved in the cluster based on the biological function and the degree of the nodes [22-24].

### Pathway enrichment and gene ontology annotation analysis

The gene ontology annotation study, including BP, cellular component (CC), and molecular function (MF) analyses, is commonly performed for mining the data obtained from high-throughput techniques [25]. In the present study, for illustrating pathways and BPs that were considerably dysregulated by the main modules-genes, the Reactome online database (https://reactome.org/) [26,27] and the Database for Annotation, Visualization and Integrated Discovery (DAVID) v6.8 (https://david.ncifcrf.gov/) [28] were utilized, respectively. The DAVID database was also used to expose CCs and MFs affected by DEGs. The significance of the signaling pathways and BPs were considered with the false discovery rate (FDR) less than 0.05 and the minimum enriched gene number of 2.

### GRN construction

The iRegulon plugin within the Cytoscape was employed for the possible detection of TFs responsible for the regulation of hub genes. A complicated approach named “genome-wide ranking-and-recovery approach” is applied by iRegulon to illustrate the motifs, consisting of a set of genes and their common regulator. Each regulator's normalized enrichment score (NES) was also determined, reflecting the associated motif's significance [29-31].

### Consensus sequences Logos and matching scores for binding sites of TFs

Using the JASPAR database (https://jaspar.genereg.net/) [32], the consensus sequences Logos of binding sites for TFs in this study was provided. Thereafter, a Position-Specific Scoring Matrix (PSSM) was constructed from the Position Frequency Matrix of the nucleotides within the TFs binding sites. A PSSM is described as a table that consists of probability information of nucleotides or residues at each position of an un-gapped multiple sequence alignment. The PSSM can be used to calculate how well a unique target sequence fits into the sequence group [33]. The total match scores for the consensus sequences of TFs were calculated manually using the R programming (version 4.0.0) [34] based on the method described by Xiong [33]. The scores can be interpreted as the probability of the consensus sequences fitting the binding sites of TFs as 2 ^(match score)^ times more likely than by random chance.

### Survival analysis

The prognostic role of the top-ranked hub genes and their salient master regulators was studied in several types of cancers using the Gene Expression Profiling Interactive Analysis 2 (GEPIA2) database [35]. The GEPIA web server was developed in 2016 for re-analyzing the cancer-related RNA sequencing resources including the Cancer Genome Atlas [36] and Genotype-Tissue Expression [37] databases. The GEPIA diminishes the differences between the two data sources by using the UCSC Xena project (http://xena.ucsc.edu/), leading to more significant and reliable results including differential expression analysis in cancerous patients compared to the healthy controls, correlation analysis, similar gene identification, as well as survival analysis. The Kaplan-Meier curves and log-rank test were used to evaluate the prognostic impact of the hub genes and major TFs in a total of 33 different cancers in the GEPIA2 database. The curves with the statistics of Log-rank test p-value and hazard ratio (HR) p-value at 0.05 were considered as significant.

### Identifying common DEGs, hub genes, and TFs in three subtypes of SGC

Three different datasets were selected from the GSE153283 as follows: one of them consisted of ACC (n = 11) and adjacent normal tissues (n = 11), the second one was a cohort composed of MEC (n = 14) and corresponding normal area (n = 14), and the third dataset included nine paired SDC and the adjacent healthy tissues. Each dataset was analyzed separately using the GEO2R tool, followed by the Cytoscape and its plugins to identify the common DEGs and hub genes in three different PIMs associated with three SGC subtypes and TFs regulating the hub genes. The cutoff points for DEGs were set to Padj < 0.001 and |Log2 FC| > 1. Moreover, the genes with the degree and betweenness centralities more than twice the average of the PIMs nodes were considered hub genes associated with each SGC subtype. Furthermore, TFs with NES > 4 were regarded as significant master regulators of the hub genes.

### Validating the results using an independent dataset

Another independent dataset was re-analyzed based on the methods used to analyze the primary dataset in this study to validate our findings, which may support the solid conclusion. The dataset GSE88804 [38] in the GEO database was considered for this purpose. This dataset was built by Andersson et al. [38] based on the platform GPL62244 (Affymetrix Human Gene 1.0 ST Array), including 14 ACC, two ACC xenograft, and seven normal salivary gland tissues. The ACC xenografts were excluded from the study before further analyses. DEGs in ACC tissues compared with the normal samples with criteria of the FDR less than 0.001 and |Log2 FC| as > 1 were considered statistically significant.

### Ethical approval

The current study was approved by the Ethics Committee of Hamadan University of Medical Sciences, Hamadan, Iran (ethics no. IR.UMSHA.REC.1400.315). No human/animal was used in this study.

## Results

### Identification of DEGs in SGC

The dataset GSE153283, consisting of 34 PGC samples and 34 corresponding healthy tissues, was analyzed using the GEO2R tool. Accordingly, a total of 137 DEGs (overexpressed = 87; under-expressed = 50) with the characteristics of corrected p-value < 0.001 and the value of |Log2 FC| > 1 were determined (Supplementary Table 1).

### PIM, clustering, and functional analysis

**Fig. 1.**
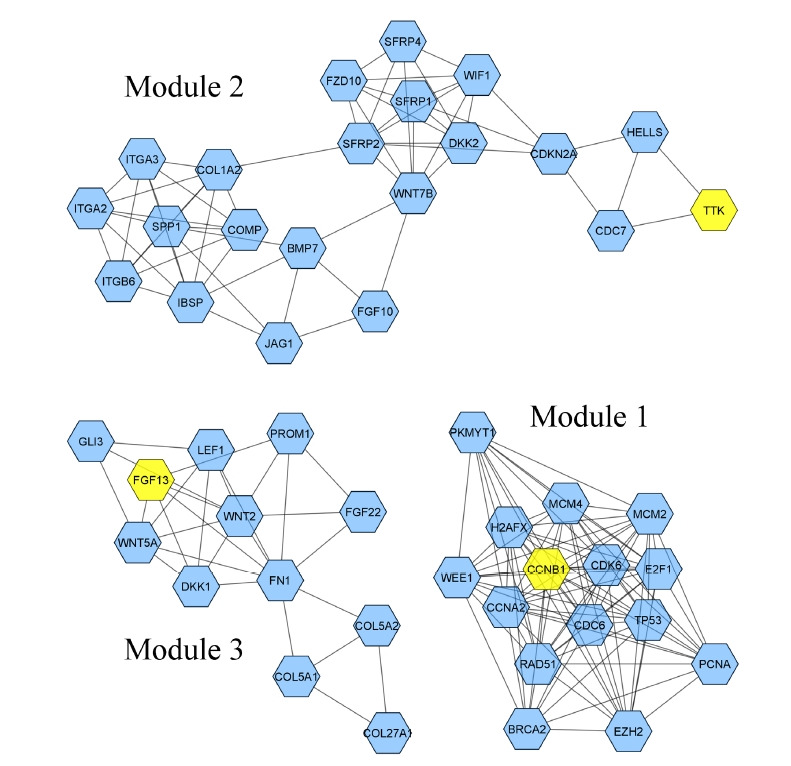
Clustering analysis via the MCODE plugin revealed three outstanding modules within the PIM. Hexagons are the genes involved in each cluster, while the yellow ones represent the seed nodes. MCODE, molecular complex detection; PIM, protein interaction map.

**Fig. 2.**
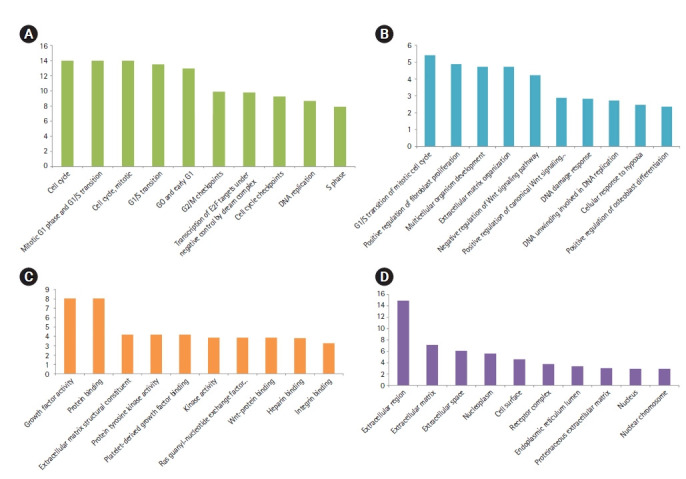
Top-ranked pathways (A), biological processes (B), molecular functions (C), and cellular components significantly affected in the primary SGC (D), based on their FDR. x-axis presents the name of the term. y-axis exhibits the –Log10 FDR. SGC, salivary gland carcinoma; FDR, false discovery rate.

**Fig. 3.**
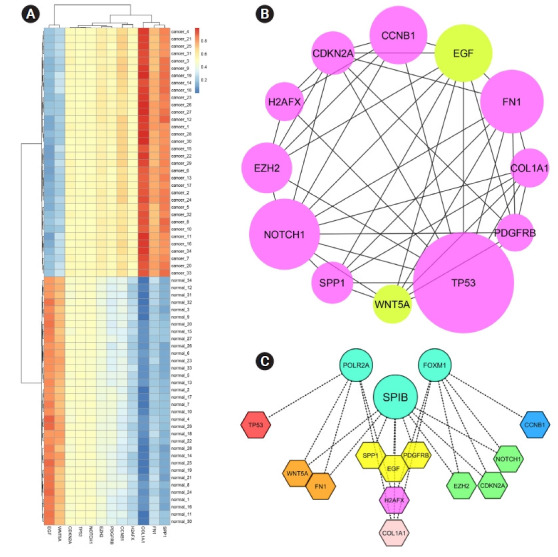
(A) Heat-map of the hub genes. The x-axis shows the hub genes, while the y-axis represents cancerous and normal observations (n = 68). Red and blue colors relatively represent a higher and lower expression of the gene in different samples after the normalization. The tissue samples from primary salivary gland carcinomas (SGCs) are well clustered from their corresponding normal tissues. (B) Interactions between hub genes. Violet and green nodes present upregulated and downregulated genes in primary SGC, respectively. The size of the nodes is positively correlated with the degree of the proteins in the main protein interaction map.. (C) Gene regulatory network analysis for hub genes. Circles represent the master regulators, while the hexagons show the hub genes: red, yellow, and blue hexagons are regulated by the *POLR2A*, *SPIB*, and *FOXM1*, respectively. *POLR2A* and *SPIB* control orange hexagons. *SPIB* and *FOXM1* regulate green hexagons. *POLR2A* and *FOXM1* regulate the purple hexagon, while the pink one is regulated by *POLR2A*, *SPIB*, and *FOXM1*.

The STRING database constructed a PIM with a confidence score of ≥0.4, based on DEGs. After ignoring the separate nodes, a network with 133 genes and 853 interactions was remained and uploaded into Cytoscape for structural analysis. The MCODE plugin demonstrated three modules with the criteria of No. of genes ≥ 10 and the MCODE score > 3, including cluster No. 1, cluster No. 2, and cluster No. 3 (Table 1, Fig. 1). These clusters were considered for further pathway and BP analyses, while CC and MF annotation analyses were performed based on DEGs. Accordingly, a total of 148 pathways, 26 BPs, 13 CCs, and 28 MFs were significantly affected in primary SGC (FDR < 0.05). All dysregulated pathways, BPs, MFs, and CCs, are listed in Supplementary Tables 2‒5, respectively. According to the functional analysis results, the most critical clusters within the PIM have principally enriched in the cell cycle-associated procedures and the regulation of fibroblast proliferation. Also, the DEGs were mainly enriched in the 'growth factor activity' (MF) and 'extracellular region' (CC) annotations (Fig. 2). Furthermore, a total of 12 nodes including *TP53*, *EGF*, *FN1*, *NOTCH1*, *EZH2*, *COL1A1*, *SPP1*, *CDKN2A*, *WNT5A*, *PDGFRB*, *CCNB1*, and *H2AFX* had a degree and betweenness centrality values more than twice the mean of the nodes inside the PIM and therefore, were suggested to be hub genes and responsible for the pathogenesis of primary SGC (Table 2). The average value of the degree and betweenness centrality was calculated to be 12.827 and 0.010266, respectively. The hierarchical clustering of the hub genes was achieved using R programming (version 4.0.0) (Fig. 3A) and the interactions between hubs were identified using the STRING database (Fig. 3B).

### Identification of master regulator

To illustrate the master regulators controlling the expression of hub genes, the iRegulon plugin was used. A total of 18 TFs with an NES > 4.0 were identified and were proposed to be statistically significant. These regulators are recommended to be essential genes in primary SGC tumorigenesis (Table 3). The results showed that the expression of all hub genes could be regulated by three TFs, including *SPIB*, *FOXM1*, and *POLR2A*: *SPIB* controls *COL1A1*, *EZH2* *PDGFRB*, *CDKN2A*, *WNT5A*, *FN1*, *NOTCH1*, *EGF*, and *SPP1*. *FOXM1* is involved in regulating *NOTCH1*, *COL1A1*, *CCNB1*, *EZH2*, *H2AFX*, and *CDKN2A*. Besides, *POLR2A* mediated the transcription of *WNT5A*, *TP53*, *H2AFX*, *FN1*, and *COL1A1* (Fig. 3C).

### Binding sites of master regulators

**Fig. 4.**
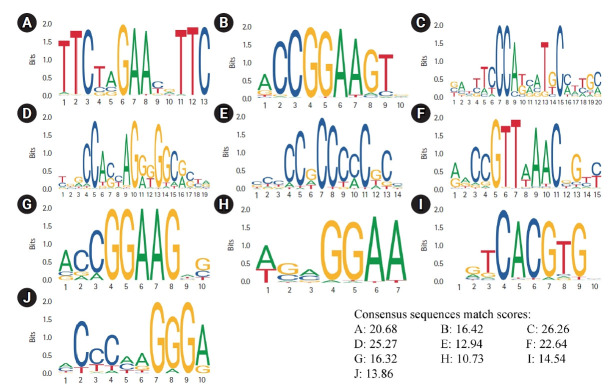
Consensus sequences Logos for the binding sites of *HSF1* (A), *ELK1* (B), *ZNF143* (C), *CTCF* (D), EGR1 (E), *MYBL2* (F), *GABPA* (G), *SPIB* (H), *ARNTL* (I), and *EBF1* (J).

A total of 18 master regulators involved in the etiology of primary SGC were searched in the JASPAR database. The binding sites Logos of 10 TFs including *HSF1*, *ELK1*, *ZNF143*, *CTCF*, EGR1, *MYBL2*, *GABPA*, *SPIB*, *ARNTL*, and *EBF1* were available in this database. The range of the binding sites matching scores was calculated between 10.73 and 25.27 for *SPIB* and *CTCF*, respectively (Fig. 4).

### Survival analysis

**Fig. 5.**
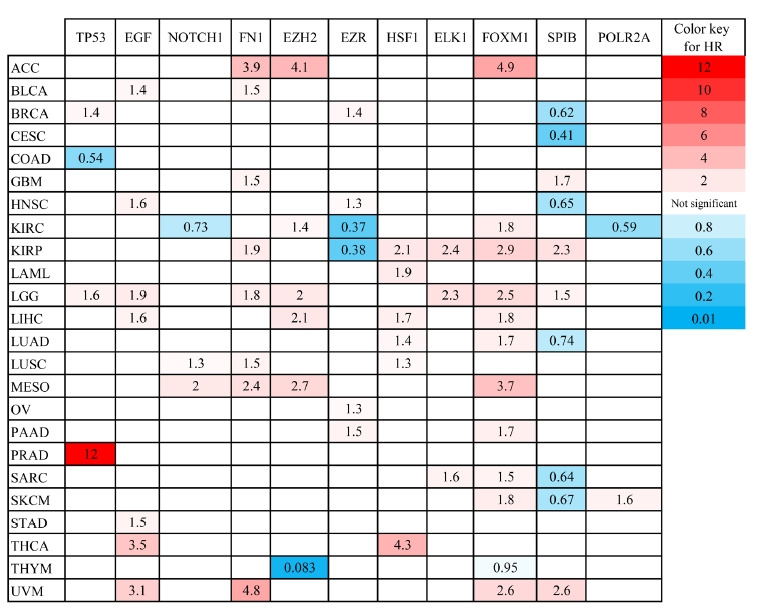
The prognostic impact of several hub genes and transcription factors involved in primary salivary gland carcinoma was studied in different cancers found in GEPIA2 database. HR, hazard ratio; ACC, adrenocortical carcinoma; BLCA, bladder urothelial carcinoma; BRCA, breast invasive carcinoma; CESC, cervical squamous cell carcinoma and endocervical adenocarcinoma; COAD, colon adenocarcinoma; GBM, glioblastoma multiform; HNSC, head and neck squamous cell carcinoma; KIRC, kidney renal clear cell carcinoma; KIRP, kidney renal papillary cell carcinoma; LAML, acute myeloid leukemia; LGG, brain lower grade glioma; LIHC, liver hepatocellular carcinoma; LUAD, lung adenocarcinoma; LUSC, lung squamous cell carcinoma; MESO, mesothelioma; OV, ovarian serous cystadenocarcinoma; PAAD, pancreatic adenocarcinoma; PRAD, prostate adenocarcinoma; SARC, sarcoma; SKCM, skin cutaneous melanoma; STAD, stomach adenocarcinoma; THCA, thyroid carcinoma; THYM, thymoma; UVM, uveal melanoma.

**Fig. 6.**
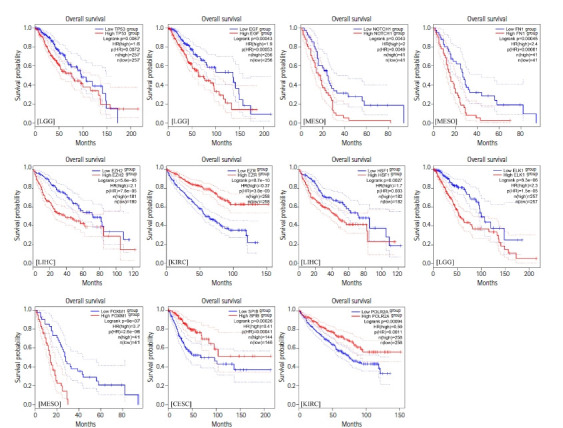
The most significant Kaplan-Meier plots illustrating the correlation between the expression of top-ranked hub genes, salient master regulators, and survival rate in several cancers. Red and blue lines demonstrate over and under-expressed genes, respectively. The x-axis and y-axis represent the survival time of cancerous patients and the probability of survival, respectively. Dotted lines show a 95% confidence interval. The cancer types are shown in the left corner of the plots. LGG, brain lower grade glioma; MESO, mesothelioma; LIHC, liver hepatocellular carcinoma; KIRC, kidney renal clear cell carcinoma; CESC, cervical squamous cell carcinoma; HR, hazard ratio.

*TP53*, *EGF*, *NOTCH1*, *FN1*, and *EZH2* demonstrated the most degree and betweenness centralities in the PIM associated with the primary SGC, and therefore, considered as top-ranked hub genes related to the etiology of the disease. Moreover, three TFs including *EZR*, *HSF1*, and *ELK1* revealed the most significant enrichment scores (NES > 5). In addition, it was found that *FOXM1*, *SPIB*, and *POLR2A* could control all the hubs' expressions. Therefore, *EZR*, *HSF1*, *ELK1*, *FOXM1*, *SPIB*, and *POLR2A* were regarded as salient regulators playing a significant role in the initiation of SGC. Survival analysis demonstrated that dysregulation of these genes is significantly associated with poor/favorable prognosis in several types of cancers with the criteria of log-rank test p-value and HR p-value < 0.05 (Fig. 5). The most significant Kaplan-Meier curves related to each gene are demonstrated in Fig. 6.

### Identifying common DEGs, hub genes, and TFs in three subtypes of SGC

**Fig. 7.**
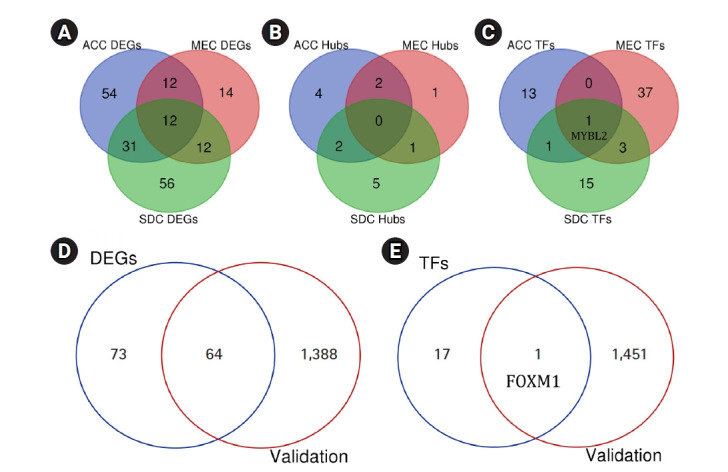
Common DEGs (A), hub genes (B), and TFs (C) between different subtypes of SGC. The number of DEGs (D) and TFs (E) found to be differentially expressed in the validation dataset GSE88804. DEG, differentially expressed gene; TF, transcription factor; SGC, salivary gland carcinoma; ACC, adenoid cystic carcinoma; MEC, mucoepidermoid carcinoma; SDC, salivary duct carcinoma.

By analyzing three datasets, each of which including one subtype of SGC and their related adjacent normal tissues, a total of 109, 50, and 111 DEGs were found in ACC, MEC, and SDC, respectively. Moreover, it was found *PRKACB*, *ETV1*, *NOTCH3*, *MAP3K1*, *BAX*, *PLCB1*, *PLCB4*, *MCM4*, *ANGPT1*, *KAT2B*, *CACNB2*, and *LIFR* were common DEGs in three subtypes of SGC (*Padj* < 0.001 and |Log2 FC| > 1). A total of eight, four, and eight genes were demonstrated salient centralities in the PIMs associated with the pathogenesis of ACC, MEC, and SDC, respectively. However, no common hubs were found among different subtypes of SGC. Furthermore, a total of 15, 41, and 20 TFs were identified regulating the hub genes in ACC, MEC, and SDC, respectively: *MYBL2* was a common master regulator in three subgroups. All DEGs, hub genes, and TFs associated with different SGC subtypes are listed in Table 4. Moreover, the common DEGs, hubs, and TFs between SGC subtypes were identified using interactive Venn diagrams (https://bioinformatics.psb.ugent.be/webtools/Venn/) (Fig. 7A‒7C).

### Validating the results

A total of 1,452 genes, including 681 upregulated and 771 downregulated genes, were found to be differentially expressed in ACC tissues compared to healthy controls (Padj < 0.001 and |Log2 FC| > 2). This was achieved by analyzing the independent dataset GSE88804 using the GEO2R tool. All these DEGs are listed in Supplementary Table 6. A total of 64 genes were found to be differentially expressed in both GEO datasets including GSE153283 and GSE88804 (Padj < 0.001 and |Log2 FC| > 1) (Table 5). Seven of which including *TP53*, *WNT5A*, *NOTCH1*, *EZH2*, *CCNB1*, *EGF*, and *PDGFRB* were previously indicated as hub genes within the PIM network associated with the primary SGC. *FOXM1*, as one of the major regulators of the hub genes in primary SGC, was also found to be significantly overexpressed in the validation dataset with the criteria of Padj = 0.00000496 and FC = 2.07. Our study's validated DEGs and TFs are shown as Venn diagrams among two datasets including GSE153283 and GSE88804 (Fig. 7D and 7E).

## Discussion

SGC has been reported to be the sixth most typical type of malignancy in the head and neck region, although said to be rare in its occurrence [39]. The exact underlying pathogenesis of the disease remains unknown [5]. In the present study, the dataset GSE153283, containing over 700 genes involved in the cancer-associated pathways, was re-analyzed to explore the primary genes within the PIM associated with the pathogenesis of primary SGC. A total of 137 genes were identified that were considerably deregulated in the cancer tissue of patients with primary SGC compared with the adjacent normal area (FDR < 0.001; |Log2 FC| > 1). Twelve of which demonstrated considerable centrality in the PIM of the disease. The top-ranked genes based on the degrees were *TP53*, *EGF*, *NOTCH1*, *FN1*, and *EZH2*, while *TP53*, *EGF*, *FN1*, *NOTCH1*, and *EZH2* were revealed to have the most betweenness centrality in the PIM, respectively.

According to the present results, it was found that *TP53* was overexpressed in the cancerous tissue of patients with SGC compared to normal adjacent tissue (Padj = 1.00e-8; Log2 FC = 1.12). Suzuki [40] performed a study to examine the expression of *TP53* in a series of 22 primary human SGC. This was done using a combination of molecular and immunohistochemical approaches, including polymerase chain reaction single-strand conformation polymorphism, direct gene sequencing, and p53 protein immunostaining. Moreover, the authors studied the correlation between the aberrant expression of p53 and genetic instability, DNA aneuploidy, and tumor growth characteristics in SGC. Suzuki [40] reported that the elevated expression of p53 was significantly associated with genetic instability in human SGC cells. According to previous studies and the current results, it may be supposed that the enhanced p53 expression in patients with primary SGC may be due to the response of increased tumor mass, which may lead to DNA instability in SGC, although this requires confirmation.

According to the present results, neurogenic locus notch homolog protein 1 (the protein expressed by the *NOTCH1*) was elevated in the cancerous tissue of primary SGC patients compared with the adjacent normal tissue (Padj = 1.84e-6; Log2 FC = 1.26). Previous studies have reported that the *CRTC1*/*MAML2* fusion protein induces the activation of the Notch signaling pathway in the MEC, resulting in enhanced cell proliferation and tumor development [41]. Moreover, it has been demonstrated that mutation in the *NOTCH1* gene occurs in 20% of patients with ACC, leading to the aggressive form of the disease that induces bone and liver metastases, resulting in a worse survival rate than a wild-type population [42].

Our results showed significantly enhanced fibronectin expression, a protein expressed by the *FN1*, in the cancerous tissue of patients with SGC compared with normal adjacent tissue (Padj = 1.48e-6; Log2 FC = 2.94). Fibronectin belongs to glycoproteins of the extracellular matrix (ECM) and is primarily synthesized by the liver [43]. Abnormal expression of the ECM components such as hyaluronan, laminin, and fibronectin may lead to cancer pathogenesis. In a previous study, Leivo et al. [44] performed a cDNA array analysis of gene expression in 13 cases of SGC, including MEC, ACC, and SDC. The authors reported that a total of five genes were overexpressed in all three subtypes of SGC, including fibronectin.

The *EZH2* gene was found to be overexpressed in the cancerous tissue of patients with primary SGC compared with the normal adjacent tissue (Padj = 2.61e-10; Log2 FC = 1.75). Zhou et al. [45] studied the effect of *EZH2* inhibitors, including *GSK126* and *EPZ6438*, on the antigen presentation in head and neck squamous cell carcinoma (HNSCC) cell lines of humans and mice. Zhou et al. [45] reported that the expression of MHC Class I was increased in human papillomavirus (HPV)-negative HNSCC lines and in the mouse models, leading to enhanced CD8+ T cell proliferation and more *interferon-gamma release*. Zhou et al. [45] reported that *EZH2* inhibition diminished the H3 methylation on the promoter of β−2-microglobulin, resulting in tumor growth suppression accompanied by anti‒programmed death-1 receptor-based therapies. However, there is still much that needs to be found about the exact function of *EZH2* in SGC etiology.

Mirsha [46] studied the correlation between monocytes/macrophages and HNSCC cells. They demonstrated that the M2-like macrophages secrete *EGF*, leading to increased motility and migration of HNSCC cells by increasing the invasive formation. Also, *EGF* induced C-C motif chemokine ligand 2 expression in HNSCC cells, resulting in the transformation of monocytes into M2-like macrophages and creating a positive feedback paracrine loop. Mirsha [46] concluded that disrupting the link between monocytes/macrophages and cancerous cells may be helpful in therapeutic aims in HNSCC. Based on our results, *EGF* was significantly decreased in the cancerous tissue of primary SGC patients compared to the adjacent healthy tissue (Padj = 8.21e-12; Log2 FC = ‒3.16). However, the exact function of *EGF* in primary SGC is less clear and requires more examination in the future to realize its role in the etiology of SGC.

Furthermore, GRN analysis demonstrated that a total of 18 TFs are participating in the control of the hub genes expression with an NES > 4.0. Besides, all the hub genes can be controlled by three master regulators, including *SPIB*, *FOXM1*, and *POLR2A*. Du et al. [47] reported that *SPIB* is commonly upregulated in human lung cancer tissues and is linked to a dismal outcome in human lung cancer. In another study, Zhang et al. [48] demonstrated that *SPIB* is an essential TF that elevates the expression of *SNAP47* in lung cancer, leading to enhanced autophagy-mediated anoikis resistance, suggesting that *SPIB* can be considered as a potential drug target for the prevention and therapeutic procedures of metastatic lung cancers. In contrast, Wood et al. [50] reported that *SPIB* was significantly overexpressed in HPV(+) HNSCC patients compared to HPV(‒) HNSCC patients. Of note, the HPV (+) HNSCC patients had a better 3- and 5-year survival than HPV (‒) HNSCC patients [50].

Liu et al. [51] demonstrated that inhibition of *POLR2A* via α-amanitin or small interfering RNAs exclusively diminished proliferation, survival, and tumorigenesis in colorectal cancer cells with hemizygous *TP53* loss. Therefore, they concluded that *POLR2A* inhibition is lethal in human cells and could be considered a novel treatment approach for human cancers [51].

Roh et al. [52] demonstrated that *FOXM1* contributes to cell cycle promoting function in HNSCC cell lines and is responsible for cell proliferation and metastasis of HNSCC cells. Also, shRNA-mediated *FOXM1* knockdown led to the diminished proliferation and reduced cell migration in three-dimensional culture and xenograft tumor models.

According to the present results, three considerable clusters within the PPI network (cluster No. 1, cluster No. 2, and cluster No. 3) were significantly involved in signaling pathways and BPs associated with tooth decay. A total of 48 genes took part in those clusters that were principally enriched in ‘cell cycle (Pathway),’ ‘Mitotic G1 phase and G1/S transition (Pathway),’ ‘G0 and Early G1 (Pathway),’ ‘G2/M Checkpoints (Pathway),’ ‘DNA replication (Pathway),’ ‘G1/S transition of mitotic cell cycle (BP),’ ‘positive regulation of fibroblast proliferation (BP),’ ‘multicellular organism development (BP),’ ‘extracellular matrix organization (BP),’ ‘negative regulation of Wnt signaling pathway (BP),’ ‘positive regulation of canonical Wnt signaling pathway (BP),’ ‘DNA damage response (BP),’ ‘DNA unwinding involved in DNA replication (BP),’ ‘cellular response to hypoxia (BP),’ and ‘positive regulation of osteoblast differentiation (BP).’

Piechocki et al. [53] studied the effects of TXT, a chemotherapy agent, on spontaneous murine salivary carcinoma. The authors reported that TXT functions as an antitumor agent through several mechanisms, including cell cycle arrest at G2/M and promoting Fas-mediated apoptosis, leading to proliferation suppression. Therefore, it may be speculated that the genes involved in the cell cycle process may be considered as potential therapeutic targets in SGC. Of note, previous studies have indicated that vitamin D contributes to targeting several cell cycle regulating factors at G1/G0 and G2/M, leading to cell cycle arrest [54].

Fibroblasts and myofibroblasts, known as cancer-associated fibroblasts (CAFs), participate in cancer-stromal interaction through different mechanisms, including efferent and afferent pathways [55]. Previous studies have shown that several paracrine and autocrine mediators such as cytokines, interleukins, keratinocyte growth factor, and TGF-β take part in the efferent pathway, leading to enhanced tumor development and formation of invasive cells from non-invasive ones [56-60]. Besides, the interaction between cancerous cells and *CAFs* in the microenvironment induces many intracellular signaling pathways resulting in increased cancer cell motility and metastasis [55-62]. Moreover, fibroblast growth factors (FGFs) consist of at least 16 identical members that mediate in various biological procedures such as cellular proliferation, chemotaxis, angiogenesis, and tissue repair. Previous studies have shown high levels of *FGF-1* in the heart, kidney glomeruli, as well as the urothelium and placenta. It has also been demonstrated that malignant salivary gland tumors and SGC cells contain high levels of *FGF-1* [63-76]. Therefore, it may be suggested that fibroblasts and FGFs take part in the primary SGC. It is worth mentioning that *FGF-13* was found to be the seed nod of module No. 3. Furthermore, ‘growth factor activity’ and ‘extracellular region’ were the most significant MF and CC categories that are affected in SGC.

Our study had several limitations, which are as follows: only patients from the University Hospital of Cologne, Germany, were included in the GSE153283 dataset, and therefore, the current results may not fully translate to patients of other nationalities. Furthermore, only 68 tissue samples were included in the GSE153283 dataset, and therefore, the sample size was not large. Last but not certainly least, a set of genes selected in the GSE153283 dataset was based on the GPL19956 platform, which does not symbolize all the genes. In future efforts, experimental approaches with large sample sizes are necessary to validate our outcome.

A total of 137 genes were recognized in the current study to be differentially expressed in the cancerous tissue of primary SGC patients compared to adjacent healthy tissues (FDR < 0.001; |Log2 FC| > 1). Moreover, *TP53*, *EGF*, *FN1*, *NOTCH1*, *EZH2*, *COL1A1*, *SPP1*, *CDKN2A*, *WNT5A*, *PDGFRB*, *CCNB1*, and *H2AFX* were considered as hub genes in the PIM associated with the primary SGC. Also, it was illustrated that SPIB, FOXM1, and POLR2A could regulate all the hub genes. Therefore, as mentioned earlier, we propose that the genes sthat participate in the etiology of primary SGC may be useful biomarkers in the therapeutic aims of primary SGC. Also, the clustering study demonstrated that the most outstanding modules within the PIM were predominantly enriched in the cell cycle-associated pathways and fibroblast proliferation process. *MYBL2* was a common TF regulating the hub genes related to the etiology of ACC, MEC, and SDC. A total of 64 DEGs and *FOXM1* were validated by analyzing an independent dataset GSE88804. However, the valuable outcome from the present study needs further research to confirm our data and demonstrate the exact role of the hub genes and their corresponding regulators in primary SGC.

## Figures and Tables

**Table 1.** A total of three considerable modules were found within the PIM via the MCODE plugin

Cluster no.	Score	No. of nodes	No. of edges	Seed node	Seed degree	Betweenness	Genes
1	13.571	15	95	*CCNB1*	29	0.0236	*TP53, CCNB1, BRCA2, PKMYT1, MCM4, CDC6, PCNA, H2AFX, EZH2, MCM2, WEE1, RAD51, E2F1, CCNA2, CDK6*
2	5.9	21	59	*TTK*	18	0.00144	*ITGB6, COMP, ITGA3, ITGA2, SPP1, WIF1, JAG1, CDKN2A, COL1A2, FGF10, SFRP2, SFRP4, BMP7, IBSP, SFRP1, DKK2, WNT7B, FZD10, HELLS, TTK, CDC7*
3	5.091	12	28	*FGF13*	20	0.0132	*COL5A1, FGF22, COL5A2, FN1, PROM1, GL13, COL27A1, FGF13, WNT2, DKK1, LEF1, WNT5A*

PIM, protein interaction map; MCODE, molecular complex detection.

**Table 2.** A total of 12 hub genes were detected in the PIM correlated with the etiology of primary SGC with the criteria of degree and betweenness more than the average of the vertexes within the network

Gene ID	Degree	Betweenness
*TP53*	70	0.287
*EGF*	49	0.104
*FN1*	44	0.098
*NOTCH1*	49	0.097
*EZH2*	35	0.056
*COL1A1*	27	0.049
*SPP1*	29	0.032
*CDKN2A*	30	0.030
*WNT5A*	27	0.026
*PDGFRB*	26	0.025
*CCNB1*	29	0.024
*H2AFX*	27	0.022

PIM, protein interaction map; SGC, salivary gland carcinoma.

**Table 3.** A total of 18 transcription factors controlling the regulation of hub genes, identified by the GRN study

TF	NES	No. of targets	Target genes
*EZR*	5.27	5	*NOTCH1, FN1, COL1A1, PDGFRB, WNT5A*
*HSF1*	5.18	5	*NOTCH1, FN1, COL1A1, PDGFRB, WNT5A*
*ELK1*	5.09	5	*NOTCH1, FN1, COL1A1, PDGFRB, WNT5A*
*FOXM1*	4.9	6	*NOTCH1, COL1A1, CCNB1, EZH2, H2AFX, CDKN2A*
*EXOSC3*	4.88	4	*FN1, COL1A1, PDGFRB, WNT5A*
*ETFB*	4.74	4	*NOTCH1, COL1A1, WNT5A, PDGFRB*
*ANXA11*	4.7	5	*WNT5A, PDGFRB, NOTCH1, COL1A1, FN1*
*ZNF143*	4.65	3	*WNT5A, PDGFRB, NOTCH1*
*CTCF*	4.64	3	*TP53, COL1A1, PDGFRB*
*EGR1*	4.58	4	*PDGFRB, WNT5A, COL1A1, EZH2*
*MYBL2*	4.57	4	*FN1, H2AFX, EZH2, CCNB1*
*GABPA*	4.45	8	*COL1A1, EZH2, PDGFRB, CDKN2A, WNT5A, FN1, NOTCH1, TP53*
*SPIB*	4.45	9	*COL1A1, EZH2, PDGFRB, CDKN2A, WNT5A, FN1, NOTCH1, EGF, SPP1*
*ARNTL*	4.28	8	*FN1, COL1A1, EZH2, NOTCH1, EGF, PDGFRB, H2AFX, WNT5A*
*POLR2A*	4.14	5	*WNT5A, TP53, H2AFX, FN1, COL1A1*
*EBF1*	4.13	5	*COL1A1, WNT5A, H2AFX, PDGFRB, FN1*
*AFF4*	4.03	3	*COL1A1, PDGFRB, NOTCH1*
*AVEN*	4.01	4	*PDGFRB, WNT5A, COL1A1, NOTCH1*

GRN, gene regulatory analysis; TF, transcription factor; NES, normalized enrichment score.

**Table 4.** Differentially expressed genes, hubs, and upstream regulators in different subtypes of SGC

SGC subtypes	Total	Gene
Differentially expressed genes in SGC subtypes		
ACC, MEC, and SDC	12	*PRKACB, ETV1, NOTCH3, MAP3K1, BAX, PLCB1, PLCB4, MCM4, ANGPT1, KAT2B, CACNB2, LIFR*
ACC and MEC	12	*PRKAR2B, CDK6, CALML5, GLI3, EGF, DUSP6, EIF4EBP1, NOTCH1, PPARG, RET, MAPT, BMP7*
ACC and SDC	31	*HIST1H3B, RASAL1, PPARGC1A, CHEK1, AMH, FZD7, FANCA, CCNB1, COMP, MLLT3, E2F1, CCNA2, CDC25B, EPHA2, MCM2, FUT8, FOXO4, PKMYT1, WNT5A, EFNA3, BRIP1, TMPRSS2, STMN1, UBE2T, WEE1, TIAM1, BMPR1B, FGF10, EZH2, MECOM, MYB*
MEC and SDC	12	*COL5A1, COL3A1, COL11A1, COL5A2, CNTFR, WNT7B, INHBA, COL1A2, COL1A1, SPRY2, PDGFC, FN1*
ACC	54	*JAG2, PRKAR1B, TNFSF10, TCF3, BNIP3, BCL2, PRKAA2, TSLP, CASP7, TP53, TCF7L1, E2F5, PIK3CB, IDH2, FGF13, ITGA2, FGFR1, IRS1, BAMBI, CD40, HELLS, LTBP1, LAMC2, INHBB, COL27A1, FGF22, LRP2, HSPA2, NPM2, FLNA, LFNG, ETV4, JAG1, PAK7, NUPR1, ID4, TLX1, RUNX1, B2M, POLD4, CTNNB1, CACNB3, FGF12, IL20RA, SIX1, PRDM1, SHC4, CCND1, RUNX1T1, ARNT2, BIRC3, IL15, MCM7, CD14*
MEC	14	*LAMB3, SFN, ID1, RPS6KA5, FGFR3, ITGA3, FGF11, LAMA5, PIK3R1, DLL1, CREB3L4, GHR, PBX1, DUSP4*
SDC	56	*WIF1, IGFBP3, CALML3, BAIAP3, PLA2G4E, WT1, PTTG2, XPA, ACVR1B, PLAU, WNT5B, PDGFRB, BIRC7, FGFR4, VEGFA, C19orf40, TGFB1, BMP8A, MYD88, PTCH1, FEN1, HIST1H3H, RAD51, H2AFX, BRCA2, ITGB6, SMAD9, TTK, CCNE2, FGFR2, DKK2, PCNA, KIT, PLD1, CDC6, SFRP1, DUSP10, MAML2, SPP1, ZIC2, IBSP, DNMT1, SPRY4, IDH1, PROM1, OSM, PRLR, DLL4, PAK3, GNG7, ITGA6, PRKDC, IL11RA, WNT2, POLE2, HIST1H3G*
Hub genes in SGC subtypes		
ACC and MEC	2	*EGF, NOTCH1*
ACC and SDC	2	*CCNB1, EZH2*
MEC and SDC	1	*FN1*
ACC	4	*CCND1, FGFR1, CTNNB1, TP53*
MEC	1	*PIK3R1*
SDC	5	*PDGFRB, VEGFA, CHEK1, BRCA2, CCNA2*
Upstream regulators in SGC subtypes		
ACC, MEC, and SDC	1	*MYBL2*
ACC and SDC	1	*FOXM1*
MEC and SDC	3	*SIN3A, *ARNTL*, JUND*
ACC	13	*HOXA7, CCDC25, POU3F1, NFATCA, SOX9, ZNF236, RFX5, MEF2A, POLI PML, TFAP2A, LHX2, E2F1*
MEC	37	*BRCA1, BARHL1, *ZNF143*, ABCF2, C19orf25, HOXB13, HNRNPA1, MAPK1, HINFP, MBD4, ADARB1, NFATC1, SMC3, TP53, MAZ, CEBPD, ATF3, POU5F1, TBP, RAD21, MAP4K2, NFKB1, ZNF362, PAX2, BATCH1, DAB2, MYC, IRF2, MAX, POLR2A, FAM48A, HOXA13, RFX1, CHD1, SNAI2, HDAC2, PDX1*
SDC	15	*ETS1, STAT3, KIAA0907, *GABPA*, CHURC1, TFDP3, MZF1, TFDP1, MXI1, TAF1, ZNF652, EXOSC3, TCEAL6, E2F4, SPIC*

SGC, salivary gland carcinoma; ACC, adenoid cystic carcinoma; MEC, mucoepidermoid carcinoma; SDC, salivary duct carcinoma.

**Table 5.** A total of 64 DEGs were common in two datasets analyzed by GEO2R

Datasets	Total	Genes
The primary dataset (GSE153283) and the dataset used for validation (GSE88804)	64	CCNB1*, CDC25B, EPHA2, CACNB3, EZH2*, HIST1H3B, SMAD9, CNTFR, CDC6, PPARG, JAG1, NUPR1, SPRY2, MAPT, RUNX1, TIAM1, BNIP3, LEF1, CDK6, PDGFRB*, EGF*, PCNA, WNT5A*, BMP7, FGF12, RUNX1T1, WIF1, PRKACB, E2F5, CCNA2, FUT8, STMN1, CDC7, ANGPT1, UBE2T, WEE1, PRKAA2, BRCA2, DDIT4, DUSP6, PLCB1, PLCB4, BRIP1, LIFR, HELLS, LRP2, EIF4EBP1, MCM4, CACNB2, FGF10, MECOM, TP53*, LAMC2, EFNA3, SIX1, BMPR1B, ETV1, NOTCH3, TTK, COL27A1, NOTCH1*, KAT2B, ARNT2, MYB

DEG, differentially expressed gene. The asterisks (*) demonstrate the genes that were considered as hubs in the protein-protein interaction network associated with the etiology of primary salivary gland carcinoma.

## Supplementary Materials

Supplementary Table 1. A total of 137 DEGs in the cancerous tissue of patients with primary SGC compared to the adjacent normal region

Supplementary Table 2. Pathways significantly affected in primary SGC

Supplementary Table 3. Biological process annotations significantly deregulated in primary SGC

Supplementary Table 4. Molecular function annotations significantly deregulated in primary SGC

Supplementary Table 5. Cellular component annotations significantly deregulated in primary SGC

Supplementary Table 6. A total of 1452 genes were differentially expressed in ACC tissues compared to the healthy controls. These results were achieved by analyzing the dataset GSE88804 which was used for validating the results obtained by analyzing the dataset GSE153283
