# Supplementary material for: Potential biomarkers and signaling pathways associated with the pathogenesis of primary salivary gland carcinoma: a bioinformatics study
Source: Genomics Inform. 2021 Dec 31;19(4):e42. doi: 10.5808/gi.21052 (PMC8752977; doi:10.5808/gi.21052)
Supplement: Supplementary Table 1. — A total of 137 DEGs in the cancerous tissue of patients with primary SGC compared to the adjacent normal region [file gi-21052suppl1.pdf]

Supplementary Table 1. A total of 137 DEGs in the cancerous tissue of patients with primary SGC compared to the adjacent normal region.

**A, Upregulated**			
**Gene ID**	**FDR**	**Log2 FC**	**|Log2FC|**
MCM4	1.20E-15	1.487	1.487
BAX	2.41E-13	1.155144	1.155144
UBE2T	6.06E-13	2.410336	2.410336
BMP7	5.87E-11	2.906639	2.906639
CCNA2	5.87E-11	1.789121	1.789121
CACNB3	5.87E-11	1.37928	1.37928
WNT7B	1.43E-10	2.367531	2.367531
NOTCH3	1.65E-10	1.434252	1.434252
CCNB1	2.00E-10	2.008264	2.008264
COL5A1	2.61E-10	2.932127	2.932127
EZH2	2.61E-10	1.747664	1.747664
STMN1	3.55E-10	2.293054	2.293054
HIST1H3B	4.96E-10	2.927563	2.927563
TIAM1	4.96E-10	1.331819	1.331819
COMP	1.19E-09	4.666134	4.666134
PCNA	1.23E-09	1.115073	1.115073
E2F5	3.49E-09	1.039199	1.039199
GLI3	4.10E-09	1.130191	1.130191
CDK6	4.58E-09	1.337196	1.337196
COL1A1	5.78E-09	4.332268	4.332268
JAG1	5.78E-09	1.562518	1.562518
CDC25B	6.56E-09	1.230128	1.230128
PKMYT1	8.42E-09	1.920141	1.920141
EFNA3	8.94E-09	1.460894	1.460894
TP53	1.00E-08	1.124664	1.124664
BRIP1	1.25E-08	1.718554	1.718554
FANCA	1.25E-08	1.619348	1.619348
HELLS	1.50E-08	1.265842	1.265842
CDC6	1.76E-08	1.356333	1.356333
MCM2	2.32E-08	1.1647	1.1647
HIST1H3G	2.41E-08	2.453365	2.453365
AMH	2.47E-08	1.85014	1.85014
LEF1	2.52E-08	1.682959	1.682959
COL11A1	2.98E-08	3.033123	3.033123
COL5A2	3.90E-08	2.650349	2.650349
RAD51	6.41E-08	1.004504	1.004504
INHBA	7.25E-08	1.911665	1.911665
HIST1H3H	8.46E-08	1.783177	1.783177

BMPR1B	9.44E-08	2.119667	2.119667
LAMA5	1.64E-07	1.136438	1.136438
H2AFX	1.69E-07	1.049835	1.049835
BRCA2	1.82E-07	1.301464	1.301464
E2F1	2.02E-07	1.398154	1.398154
ITGA3	2.05E-07	1.303635	1.303635
COL1A2	3.12E-07	2.941172	2.941172
LFNG	4.93E-07	1.081095	1.081095
RUNX1	8.35E-07	1.031207	1.031207
LAMC2	9.82E-07	1.599252	1.599252
BMP8A	1.02E-06	1.742207	1.742207
SPP1	1.07E-06	3.203717	3.203717
NEG_F	1.07E-06	1.209753	1.209753
CDKN2A	1.30E-06	1.403393	1.403393
FN1	1.48E-06	2.937004	2.937004
PLAT	1.48E-06	1.669762	1.669762
NOTCH1	1.84E-06	1.257412	1.257412
COL3A1	1.92E-06	2.922758	2.922758
WEE1	2.17E-06	1.304569	1.304569
LTBP1	2.75E-06	1.016971	1.016971
TTK	4.63E-06	1.461394	1.461394
ARNT2	7.61E-06	1.264946	1.264946
GAS1	7.86E-06	1.303616	1.303616
MYB	8.80E-06	2.23375	2.23375
ITGB6	9.19E-06	2.125866	2.125866
WNT2	1.30E-05	1.372551	1.372551
ITGA2	1.31E-05	1.326669	1.326669
IGFBP3	1.44E-05	1.414448	1.414448
PPP2R2C	1.63E-05	1.048263	1.048263
IL11	2.45E-05	1.360118	1.360118
SFRP2	2.63E-05	2.554643	2.554643
FZD10	3.51E-05	1.758787	1.758787
COL27A1	3.86E-05	1.707634	1.707634
IBSP	4.03E-05	1.394282	1.394282
FGFR4	4.12E-05	1.282904	1.282904
FAS	4.34E-05	1.058873	1.058873
DKK1	4.55E-05	1.191287	1.191287
DKK2	4.59E-05	1.239346	1.239346
FGF11	5.15E-05	1.092554	1.092554
DUSP4	6.22E-05	1.661091	1.661091
PLAU	6.85E-05	1.778566	1.778566
CDC7	7.68E-05	1.114727	1.114727
PDGFRB	1.36E-04	1.093068	1.093068

DDIT4	2.71E-04	1.189378	1.189378
ZIC2	2.84E-04	1.245776	1.245776
IL2RA	3.51E-04	1.428151	1.428151
LIF	3.86E-04	1.729584	1.729584
SFRP4	4.10E-04	2.036291	2.036291
ETV4	7.47E-04	1.063504	1.063504
**B, Downregulated**			
**Gene ID**	**FDR**	**Log2 FC**	**|Log2 FC|**
KAT2B	4.71E-20	-2.236608	2.236608
PLCB4	2.10E-16	-3.051112	3.051112
ANGPT1	2.16E-16	-2.134726	2.134726
ETV1	3.99E-15	-3.803636	3.803636
CACNB2	6.94E-15	-2.292984	2.292984
LIFR	8.72E-15	-2.488536	2.488536
FOXO4	1.74E-14	-1.043022	1.043022
PRKACB	4.63E-14	-2.098262	2.098262
FUT8	2.33E-13	-2.167535	2.167535
MAP3K1	5.79E-13	-1.201679	1.201679
PLCB1	1.71E-12	-2.215593	2.215593
EGF	8.21E-12	-3.161763	3.161763
DUSP6	2.96E-11	-1.55409	1.55409
WNT5A	5.08E-11	-2.707979	2.707979
EIF4EBP1	1.04E-10	-1.999655	1.999655
CNTFR	1.94E-10	-1.878147	1.878147
RASAL1	2.32E-10	-2.437847	2.437847
MLLT3	3.68E-10	-1.304671	1.304671
SMAD9	6.94E-10	-1.790751	1.790751
MAPT	1.23E-09	-2.183841	2.183841
WIF1	1.46E-09	-3.909855	3.909855
FGF10	1.92E-09	-1.692443	1.692443
SIX1	2.84E-09	-2.076989	2.076989
TMPRSS2	4.64E-09	-2.233292	2.233292
RUNX1T1	5.44E-09	-1.55817	1.55817
NUPR1	1.54E-08	-1.185028	1.185028
SPRY2	1.57E-08	-1.170974	1.170974
FGF13	2.52E-08	-1.837533	1.837533
FGF12	2.56E-08	-2.12187	2.12187
BAIAP3	5.76E-08	-1.142751	1.142751
LRP2	1.14E-07	-2.032867	2.032867
BNIP3	1.24E-07	-1.224567	1.224567
PDGFC	1.31E-07	-1.003977	1.003977
CALML5	1.39E-07	-2.963915	2.963915
PPARG	2.91E-07	-1.101846	1.101846

PPARGC1A	3.62E-07	-2.156868	2.156868
PRKAA2	1.18E-06	-1.637637	1.637637
PROM1	1.23E-06	-2.000625	2.000625
HSPA2	1.37E-06	-1.387381	1.387381
FGF22	1.68E-06	-1.189451	1.189451
MECOM	2.92E-06	-1.079338	1.079338
EPHA2	4.21E-06	-1.314521	1.314521
PAK3	1.30E-05	-1.104173	1.104173
CXXC4	1.67E-05	-1.421171	1.421171
PRKAR2B	1.93E-05	-1.032897	1.032897
RET	2.04E-05	-1.28595	1.28595
SFRP1	1.26E-04	-1.586223	1.586223
FGFR2	1.56E-04	-1.635296	1.635296
MAPK8IP2	3.40E-04	-1.235597	1.235597
BIRC3	6.05E-04	-1.104916	1.104916

DEG, differentially expressed genes; SGC, salivary gland carcinoma; FDR, false discovery rate; FC, fold change.
