# Supplementary material for: Potential biomarkers and signaling pathways associated with the pathogenesis of primary salivary gland carcinoma: a bioinformatics study
Source: Genomics Inform. 2021 Dec 31;19(4):e42. doi: 10.5808/gi.21052 (PMC8752977; doi:10.5808/gi.21052)
Supplement: Supplementary Table 2. — Pathways significantly affected in primary SGC [file gi-21052suppl2.pdf]

Supplementary Table 2. Pathways significantly affected in primary SGC.

No. of Cluster	Pathway ID	Pathway name	# Entities	FDR
	R-HSA-1640170	Cell Cycle	21	1.04E-14
	R-HSA-69278	Cell Cycle, Mitotic	19	1.04E-14
	R-HSA-453279	Mitotic G1 phase and G1/S transition	15	1.04E-14
	R-HSA-69206	G1/S Transition	11	3.11E-14
	R-HSA-1538133	G0 and Early G1	8	1.12E-13
	R-HSA-69481	G2/M Checkpoints	9	1.24E-10
	R-HSA-1362277	Transcription of E2F targets under negative control by DREAM complex	6	1.65E-10
	R-HSA-69620	Cell Cycle Checkpoints	10	5.46E-10
	R-HSA-69306	DNA Replication	8	2.17E-09
	R-HSA-69242	S Phase	8	1.26E-08
	R-HSA-2559580	Oxidative Stress Induced Senescence	7	1.78E-08
	R-HSA-2559583	Cellular Senescence	8	2.35E-08
	R-HSA-6791312	TP53 Regulates Transcription of Cell Cycle Genes	6	2.56E-08
	R-HSA-453274	Mitotic G2-G2/M phases	8	3.11E-08
	R-HSA-68867	Assembly of the pre-replicative complex	6	3.11E-08
	R-HSA-69002	DNA Replication Pre-Initiation	6	1.25E-07
	R-HSA-69205	G1/S-Specific Transcription	5	1.72E-07
	R-HSA-8953750	Transcriptional Regulation by E2F6	5	2.25E-07
1	R-HSA-6804116	TP53 Regulates Transcription of Genes Involved in G1 Cell Cycle Arrest	4	5.90E-07
	R-HSA-1362300	Transcription of E2F targets under negative control by p107 (RBL1) and p130 (RBL2) in complex with HDAC1	4	5.90E-07
	R-HSA-156711	Polo-like kinase mediated events	4	9.55E-07
	R-HSA-69239	Synthesis of DNA	6	9.97E-07
	R-HSA-68949	Orc1 removal from chromatin	5	1.61E-06
	R-HSA-69478	G2/M DNA replication checkpoint	3	2.45E-06
	R-HSA-68911	G2 Phase	3	2.45E-06
	R-HSA-69273	Cyclin A/B1/B2 associated events during G2/M transition	4	2.72E-06
	R-HSA-5693532	DNA Double-Strand Break Repair	6	2.92E-06
	R-HSA-69052	Switching of origins to a post-replicative state	5	3.85E-06
	R-HSA-68962	Activation of the pre-replicative complex	4	3.85E-06
	R-HSA-69190	DNA strand elongation	4	4.17E-06
	R-HSA-176187	Activation of ATR in response to replication stress	4	4.17E-06
	R-HSA-2559585	Oncogene Induced Senescence	4	5.59E-06
	R-HSA-68689	CDC6 association with the ORC:origin complex	3	6.63E-06
	R-HSA-69275	G2/M Transition	6	7.49E-06
	R-HSA-176974	Unwinding of DNA	3	7.49E-06
	R-HSA-5693567	HDR through Homologous Recombination (HRR) or Single Strand Annealing (SSA)	5	1.17E-05
	R-HSA-5693538	Homology Directed Repair	5	1.48E-05

R-HSA-212436	Generic Transcription Pathway	12	1.58E-05
R-HSA-9661069	Defective binding of RB1 mutants to E2F1,(E2F2, E2F3)	3	2.09E-05
R-HSA-9659787	Aberrant regulation of mitotic G1/S transition in cancer due to RB1 defects	3	2.09E-05
R-HSA-73857	RNA Polymerase II Transcription	12	3.26E-05
R-HSA-6804114	TP53 Regulates Transcription of Genes Involved in G2 Cell Cycle Arrest	3	3.26E-05
R-HSA-69473	G2/M DNA damage checkpoint	4	5.29E-05
R-HSA-3700989	Transcriptional Regulation by TP53	7	6.08E-05
R-HSA-2262752	Cellular responses to stress	8	6.81E-05
R-HSA-1912408	Pre-NOTCH Transcription and Translation	4	7.63E-05
R-HSA-8953897	Cellular responses to external stimuli	8	8.18E-05
R-HSA-74160	Gene expression (Transcription)	12	8.24E-05
R-HSA-6804754	Regulation of TP53 Expression	2	1.10E-04
R-HSA-73894	DNA Repair	6	1.29E-04
R-HSA-9687139	Aberrant regulation of mitotic cell cycle due to RB1 defects	3	1.29E-04
R-HSA-9675126	Diseases of mitotic cell cycle	3	1.52E-04
R-HSA-1912422	Pre-NOTCH Expression and Processing	4	1.54E-04
R-HSA-111448	Activation of NOXA and translocation to mitochondria	2	1.98E-04
R-HSA-6806003	Regulation of TP53 Expression and Degradation	3	2.67E-04
R-HSA-69236	G1 Phase	3	3.61E-04
R-HSA-69231	Cyclin D associated events in G1	3	3.61E-04
R-HSA-912446	Meiotic recombination	3	5.27E-04
R-HSA-139915	Activation of PUMA and translocation to mitochondria	2	5.48E-04
R-HSA-2559586	DNA Damage/Telomere Stress Induced Senescence	3	9.53E-04
R-HSA-5685942	HDR through Homologous Recombination (HRR)	3	9.93E-04
R-HSA-75035	Chk1/Chk2(Cds1) mediated inactivation of Cyclin B:Cdk1 complex	2	1.23E-03
R-HSA-69202	Cyclin E associated events during G1/S transition	3	1.43E-03
R-HSA-69656	Cyclin A:Cdk2-associated events at S phase entry	3	1.43E-03
R-HSA-2559582	Senescence-Associated Secretory Phenotype (SASP)	3	1.47E-03
R-HSA-1500620	Meiosis	3	1.52E-03
R-HSA-113510	E2F mediated regulation of DNA replication	2	2.33E-03
R-HSA-157579	Telomere Maintenance	3	2.61E-03
R-HSA-157118	Signaling by NOTCH	4	2.67E-03
R-HSA-1474165	Reproduction	3	3.51E-03
R-HSA-5693554	Resolution of D-loop Structures through Synthesis-Dependent Strand Annealing (SDSA)	2	3.62E-03
R-HSA-73886	Chromosome Maintenance	3	4.87E-03
R-HSA-5693568	Resolution of D-loop Structures through Holliday Junction Intermediates	2	5.18E-03
R-HSA-114452	Activation of BH3-only proteins	2	5.18E-03
R-HSA-5693537	Resolution of D-Loop Structures	2	5.47E-03
R-HSA-5693616	Presynaptic phase of homologous DNA pairing and strand exchange	2	6.69E-03

	R-HSA-6804757	Regulation of TP53 Degradation	2	7.34E-03
	R-HSA-212300	PRC2 methylates histones and DNA	2	7.68E-03
	R-HSA-5693579	Homologous DNA Pairing and Strand Exchange	2	7.68E-03
	R-HSA-8853884	Transcriptional Regulation by VENTX	2	9.10E-03
	R-HSA-5633007	Regulation of TP53 Activity	3	1.00E-02
	R-HSA-2299718	Condensation of Prophase Chromosomes	2	1.02E-02
	R-HSA-109606	Intrinsic Pathway for Apoptosis	2	1.02E-02
	R-HSA-5693565	Recruitment and ATM-mediated phosphorylation of repair and signaling proteins at DNA double strand breaks	2	1.16E-02
	R-HSA-180786	Extension of Telomeres	2	1.23E-02
	R-HSA-69563	p53-Dependent G1 DNA Damage Response	2	1.26E-02
	R-HSA-69580	p53-Dependent G1/S DNA damage checkpoint	2	1.26E-02
	R-HSA-8943724	Regulation of PTEN gene transcription	2	1.26E-02
	R-HSA-9616222	Transcriptional regulation of granulopoiesis	2	1.30E-02
	R-HSA-69615	G1/S DNA Damage Checkpoints	2	1.33E-02
	R-HSA-69017	CDK-mediated phosphorylation and removal of Cdc6	2	1.44E-02
	R-HSA-5693606	DNA Double Strand Break Response	2	1.44E-02
	R-HSA-176409	APC/C:Cdc20 mediated degradation of mitotic proteins	2	1.48E-02
	R-HSA-176814	Activation of APC/C and APC/C:Cdc20 mediated degradation of mitotic proteins	2	1.52E-02
	R-HSA-8852276	The role of GTSE1 in G2/M progression after G2 checkpoint	2	1.75E-02
	R-HSA-176408	Regulation of APC/C activators between G1/S and early anaphase	2	1.75E-02
	R-HSA-5693607	Processing of DNA double-strand break ends	2	2.04E-02
	R-HSA-174143	APC/C-mediated degradation of cell cycle proteins	2	2.13E-02
	R-HSA-453276	Regulation of mitotic cell cycle	2	2.13E-02
	R-HSA-6804756	Regulation of TP53 Activity through Phosphorylation	2	2.26E-02
	R-HSA-73884	Base Excision Repair	2	2.45E-02
	R-HSA-5619507	Activation of HOX genes during differentiation	2	3.01E-02
	R-HSA-5617472	Activation of anterior HOX genes in hindbrain development during early embryogenesis	2	3.01E-02
	R-HSA-68875	Mitotic Prophase	2	3.01E-02
	R-HSA-212165	Epigenetic regulation of gene expression	2	3.01E-02
	R-HSA-6807070	PTEN Regulation	2	3.39E-02
	R-HSA-3108232	SUMO E3 ligases SUMOylate target proteins	2	3.84E-02
	R-HSA-109581	Apoptosis	2	4.07E-02
	R-HSA-2990846	SUMOylation	2	4.18E-02
	R-HSA-983231	Factors involved in megakaryocyte development and platelet production	2	4.26E-02
	R-HSA-5689880	Ub-specific processing proteases	2	4.75E-02
2	R-HSA-216083	Integrin cell surface interactions	7	1.08E-07
	R-HSA-8878159	Transcriptional regulation by RUNX3	7	4.35E-07
	R-HSA-2559585	Oncogene Induced Senescence	5	1.53E-06
	R-HSA-3772470	Negative regulation of TCF-dependent signaling by WNT ligand antagonists	4	1.53E-06

	R-HSA-1474244	Extracellular matrix organization	8	1.17E-05
	R-HSA-3000178	ECM proteoglycans	5	2.02E-05
	R-HSA-8853884	Transcriptional Regulation by VENTX	4	8.62E-05
	R-HSA-2559580	Oxidative Stress Induced Senescence	5	9.20E-05
	R-HSA-9675132	Diseases of cellular response to stress	2	6.15E-04
	R-HSA-9630747	Diseases of Cellular Senescence	2	6.15E-04
	R-HSA-8874081	MET activates PTK2 signaling	3	7.06E-04
	R-HSA-2559583	Cellular Senescence	5	8.79E-04
	R-HSA-8875878	MET promotes cell motility	3	1.69E-03
	R-HSA-212436	Generic Transcription Pathway	11	2.54E-03
	R-HSA-8949275	RUNX3 Regulates Immune Response and Cell Migration	2	2.54E-03
	R-HSA-73857	RNA Polymerase II Transcription	11	4.77E-03
	R-HSA-75892	Platelet Adhesion to exposed collagen	2	4.84E-03
	R-HSA-8941856	RUNX3 regulates NOTCH signaling	2	4.84E-03
	R-HSA-8951936	RUNX3 regulates p14-ARF	2	4.84E-03
	R-HSA-195721	Signaling by WNT	5	5.47E-03
	R-HSA-6806834	Signaling by MET	3	6.78E-03
	R-HSA-74160	Gene expression (Transcription)	11	7.45E-03
	R-HSA-201681	TCF dependent signaling in response to WNT	4	8.50E-03
	R-HSA-3000170	Syndecan interactions	2	1.22E-02
	R-HSA-3000157	Laminin interactions	2	1.26E-02
	R-HSA-2129379	Molecules associated with elastic fibres	2	1.77E-02
	R-HSA-1566948	Elastic fibre formation	2	2.14E-02
	R-HSA-8953750	Transcriptional Regulation by E2F6	2	2.14E-02
	R-HSA-3000171	Non-integrin membrane-ECM interactions	2	2.95E-02
	R-HSA-9006934	Signaling by Receptor Tyrosine Kinases	5	3.82E-02
3	R-HSA-8874081	MET activates PTK2 signaling	5	3.10E-08
	R-HSA-8875878	MET promotes cell motility	5	8.39E-08
	R-HSA-6806834	Signaling by MET	5	1.55E-06
	R-HSA-8948216	Collagen chain trimerization	4	5.22E-06
	R-HSA-2022090	Assembly of collagen fibrils and other multimeric structures	4	2.17E-05
	R-HSA-1650814	Collagen biosynthesis and modifying enzymes	4	2.95E-05
	R-HSA-162582	Signal Transduction	13	3.30E-05
	R-HSA-1474290	Collagen formation	4	5.93E-05
	R-HSA-3238698	WNT ligand biogenesis and trafficking	3	5.93E-05
	R-HSA-3000170	Syndecan interactions	3	5.93E-05
	R-HSA-1474244	Extracellular matrix organization	5	2.48E-04
	R-HSA-195721	Signaling by WNT	5	2.48E-04
	R-HSA-9006934	Signaling by Receptor Tyrosine Kinases	6	3.70E-04
	R-HSA-3000171	Non-integrin membrane-ECM interactions	3	3.83E-04
	R-HSA-3000178	ECM proteoglycans	3	7.39E-04

R-HSA-216083	Integrin cell surface interactions	3	9.82E-04
R-HSA-3772470	Negative regulation of TCF-dependent signaling by WNT ligand antagonists	2	9.87E-04
R-HSA-373080	Class B/2 (Secretin family receptors)	3	1.28E-03
R-HSA-1474228	Degradation of the extracellular matrix	3	3.61E-03
R-HSA-419037	NCAM1 interactions	2	7.30E-03
R-HSA-201681	TCF dependent signaling in response to WNT	3	1.07E-02
R-HSA-1442490	Collagen degradation	2	1.51E-02
R-HSA-375165	NCAM signaling for neurite out-growth	2	1.56E-02
R-HSA-4086398	Ca2+ pathway	2	1.94E-02
R-HSA-186797	Signaling by PDGF	2	1.94E-02

SGC, salivary gland carcinoma; FDR, false discovery rate.
