# Supplementary material for: Potential biomarkers and signaling pathways associated with the pathogenesis of primary salivary gland carcinoma: a bioinformatics study
Source: Genomics Inform. 2021 Dec 31;19(4):e42. doi: 10.5808/gi.21052 (PMC8752977; doi:10.5808/gi.21052)
Supplement: Supplementary Table 3. — Biological process annotations significantly deregulated in primary SGC [file gi-21052suppl3.pdf]

Supplementary Table 3. Biological process annotations significantly deregulated in primary SGC.

No. of Cluster	GO ID	Term	# Entities	FDR
1	GO:0000082	G1/S transition of mitotic cell cycle	6	3.90E-06
	GO:0048146	positive regulation of fibroblast proliferation	5	1.30E-05
	GO:0006977	DNA damage response	4	1.50E-03
	GO:0006268	DNA unwinding involved in DNA replication	3	1.90E-03
	GO:0071456	cellular response to hypoxia	4	3.40E-03
	GO:0010165	response to X-ray	3	6.00E-03
	GO:0051301	cell division	5	6.00E-03
	GO:0006260	DNA replication	4	8.50E-03
	GO:0006270	DNA replication initiation	3	8.50E-03
	GO:0000722	telomere maintenance via recombination	3	8.50E-03
	GO:0000079	regulation of cyclin-dependent protein serine/threonine kinase activity	3	1.10E-02
	GO:0007067	mitotic nuclear division	4	2.30E-02
	GO:1990426	mitotic recombination-dependent replication fork processing	2	3.20E-02
	GO:0000724	double-strand break repair via homologous recombination	3	3.20E-02
2	GO:0007275	multicellular organism development	9	1.90E-05
	GO:0030198	extracellular matrix organization	7	1.90E-05
	GO:0030178	negative regulation of Wnt signaling pathway	5	6.00E-05
	GO:0090263	positive regulation of canonical Wnt signaling pathway	5	1.30E-03
	GO:0045669	positive regulation of osteoblast differentiation	4	4.40E-03
	GO:0008285	negative regulation of cell proliferation	6	6.40E-03
	GO:0007155	cell adhesion	6	1.10E-02
	GO:0007229	integrin-mediated signaling pathway	4	1.20E-02
	GO:0043065	positive regulation of apoptotic process	5	2.00E-02
	GO:0010634	positive regulation of epithelial cell migration	3	3.30E-02
	GO:0090090	negative regulation of canonical Wnt signaling pathway	4	3.90E-02
3	GO:0090090	negative regulation of canonical Wnt signaling pathway	4	4.10E-02
	GO:0030198	extracellular matrix organization	4	4.10E-02

SGC, salivary gland carcinoma; FDR, false discovery rate..
