# Supplementary material for: Potential biomarkers and signaling pathways associated with the pathogenesis of primary salivary gland carcinoma: a bioinformatics study
Source: Genomics Inform. 2021 Dec 31;19(4):e42. doi: 10.5808/gi.21052 (PMC8752977; doi:10.5808/gi.21052)
Supplement: Supplementary Table 4. — Molecular function annotations significantly deregulated in primary SGC [file gi-21052suppl4.pdf]

**Supplementary Table 4.** Molecular function annotations significantly deregulated in primary SGC.

| GO ID | Term | Count | FDR |
|---|---|---|---|
| GO:0008083 | growth factor activity | 15 | 9.10E-09 |
| GO:0005515 | protein binding | 107 | 9.10E-09 |
| GO:0005201 | extracellular matrix structural constituent | 8 | 6.50E-05 |
| GO:0004713 | protein tyrosine kinase activity | 10 | 6.50E-05 |
| GO:0048407 | platelet-derived growth factor binding | 5 | 6.50E-05 |
| GO:0016301 | kinase activity | 12 | 1.40E-04 |
| GO:0005088 | Ras guanyl-nucleotide exchange factor activity | 9 | 1.40E-04 |
| GO:0017147 | Wnt-protein binding | 6 | 1.40E-04 |
| GO:0008201 | heparin binding | 10 | 1.60E-04 |
| GO:0005178 | integrin binding | 8 | 5.50E-04 |
| GO:0042813 | Wnt-activated receptor activity | 5 | 6.10E-04 |
| GO:0019901 | protein kinase binding | 13 | 1.00E-03 |
| GO:0003682 | chromatin binding | 13 | 1.40E-03 |
| GO:0019899 | enzyme binding | 12 | 1.40E-03 |
| GO:0046934 | phosphatidylinositol-4 | 6 | 2.40E-03 |
| GO:0046982 | protein heterodimerization activity | 13 | 5.60E-03 |
| GO:0008134 | transcription factor binding | 10 | 7.00E-03 |
| GO:0005125 | cytokine activity | 8 | 7.70E-03 |
| GO:0005524 | ATP binding | 25 | 9.40E-03 |
| GO:0003700 | transcription factor activity | 18 | 2.10E-02 |
| GO:0005109 | frizzled binding | 4 | 3.50E-02 |
| GO:0042393 | histone binding | 6 | 3.50E-02 |
| GO:0003677 | DNA binding | 25 | 3.50E-02 |
| GO:0030331 | estrogen receptor binding | 4 | 3.50E-02 |
| GO:0005160 | transforming growth factor beta receptor binding | 4 | 4.60E-02 |
| GO:0046332 | SMAD binding | 4 | 4.60E-02 |
| GO:0016303 | 1-phosphatidylinositol-3-kinase activity | 4 | 4.60E-02 |
| GO:0030971 | receptor tyrosine kinase binding | 4 | 4.60E-02 |

SGC, salivary gland carcinoma; FDR, false discovery rate.
