# Supplementary material for: Potential biomarkers and signaling pathways associated with the pathogenesis of primary salivary gland carcinoma: a bioinformatics study
Source: Genomics Inform. 2021 Dec 31;19(4):e42. doi: 10.5808/gi.21052 (PMC8752977; doi:10.5808/gi.21052)
Supplement: Supplementary Table 5. — Cellular component annotations significantly deregulated in primary SGC [file gi-21052suppl5.pdf]

Supplementary Table 5. Cellular component annotations significantly deregulated in primary SGC.

GO ID	Term	Count	FDR
GO:0005576	extracellular region	49	1.20E-15
GO:0031012	extracellular matrix	17	7.00E-08
GO:0005615	extracellular space	32	7.60E-07
GO:0005654	nucleoplasm	47	2.40E-06
GO:0009986	cell surface	18	2.40E-05
GO:0043235	receptor complex	9	1.70E-04
GO:0005788	endoplasmic reticulum lumen	10	4.10E-04
GO:0005578	proteinaceous extracellular matrix	11	8.60E-04
GO:0005634	nucleus	63	1.10E-03
GO:0000784	nuclear chromosome	8	1.10E-03
GO:0005581	collagen trimer	7	1.20E-03
GO:0005737	cytoplasm	60	2.40E-03
GO:0070062	extracellular exosome	37	8.80E-03

SGC, salivary gland carcinoma; FDR, false discovery rate.
