# Supplementary material for: Potential biomarkers and signaling pathways associated with the pathogenesis of primary salivary gland carcinoma: a bioinformatics study
Source: Genomics Inform. 2021 Dec 31;19(4):e42. doi: 10.5808/gi.21052 (PMC8752977; doi:10.5808/gi.21052)
Supplement: Supplementary Table 6. — A total of 1452 genes were differentially expressed in ACC tissues compared to the healthy controls. These results were achieved by analyzing the dataset GSE88804 which was used for validating the results obtained by analyzing the dataset GSE153283 [file gi-21052suppl6.pdf]

Supplementary Table 6. A total of 1452 genes were differentially expressed in ACC tissues compared to the healthy controls. These results were achieved by analyzing the dataset GSE88804 which was used for validating the results obtained by analyzing the dataset GSE153283.

**A, Upregulated**			
**Gene ID**	**FDR**	**Log2 FC**	**|Log2 FC|**
GABRP	4.05E-11	5.3200	5.3200
MYB	6.83E-11	5.1738	5.1738
HORMAD1	3.97E-08	4.2742	4.2742
ABCA13	2.22E-07	4.0394	4.0394
NETO2	1.43E-06	3.8245	3.8245
HAPLN1	7.01E-06	3.8151	3.8151
VCAN	1.47E-10	3.7730	3.7730
VTCN1	1.68E-11	3.7265	3.7265
ART3	1.53E-05	3.6737	3.6737
BMPR1B	8.37E-12	3.6483	3.6483
PDZK1	2.30E-04	3.5980	3.5980
TBX22	2.92E-08	3.5097	3.5097
PRAME	4.04E-11	3.4791	3.4791
FABP7	8.63E-11	3.4118	3.4118
STMN1	9.24E-13	3.2609	3.2609
OR2L8	1.13E-05	3.2488	3.2488
ZNF730	2.31E-09	3.1840	3.1840
FNDC1	2.97E-07	3.1000	3.1000
ELAVL2	3.75E-08	3.0105	3.0105
OVOS2	4.01E-07	2.9765	2.9765
SHC4	5.52E-07	2.9643	2.9643
KRT15	4.11E-05	2.9243	2.9243
OR2L2	6.89E-06	2.9240	2.9240
ANLN	6.83E-11	2.9067	2.9067
EDIL3	3.07E-06	2.9004	2.9004
GUCY1A3	1.36E-07	2.8810	2.8810
OR2AK2	1.00E-05	2.8436	2.8436
DLX5	4.36E-05	2.8406	2.8406
BRIP1	2.80E-09	2.7101	2.7101
MGC39584	4.60E-04	2.7029	2.7029
DTL	1.25E-07	2.6897	2.6897
RASGRP1	1.57E-06	2.6687	2.6687
CDK6	1.38E-10	2.6356	2.6356
RAPGEF4	4.75E-06	2.6123	2.6123

BAMBI	6.63E-05	2.5991	2.5991
HEY2	2.23E-05	2.5986	2.5986
SERPINE2	9.92E-05	2.5764	2.5764
EFHD1	1.62E-09	2.5648	2.5648
HELLS	1.11E-10	2.5499	2.5499
LAMB1	4.01E-07	2.5481	2.5481
THBS2	1.17E-06	2.5177	2.5177
ZNF286A	2.12E-10	2.5143	2.5143
NPNT	1.39E-07	2.4906	2.4906
PXDN	1.81E-06	2.4861	2.4861
VIT	2.56E-04	2.4780	2.4780
PRLR	1.53E-06	2.4762	2.4762
TOP2A	3.61E-08	2.4602	2.4602
XRCC2	1.26E-08	2.4511	2.4511
MFGE8	2.17E-07	2.4211	2.4211
EZH2	1.80E-11	2.4211	2.4211
OR2L5	4.94E-05	2.4067	2.4067
TTYH1	1.65E-07	2.4023	2.4023
SEMA6D	5.99E-07	2.3890	2.3890
JAG1	9.21E-08	2.3857	2.3857
CCNB2	5.36E-08	2.3772	2.3772
CENPF	9.65E-08	2.3509	2.3509
SYCP2	4.00E-08	2.3438	2.3438
AADAT	8.39E-07	2.3131	2.3131
COLEC12	1.21E-05	2.3063	2.3063
CENPK	1.80E-07	2.2916	2.2916
SEPT4	5.02E-08	2.2777	2.2777
NUSAP1	1.16E-07	2.2708	2.2708
ITGA9	2.86E-07	2.2700	2.2700
COL9A1	2.21E-04	2.2651	2.2651
OBP2B	7.65E-06	2.2651	2.2651
EPHA7	1.31E-05	2.2604	2.2604
ZNF300	7.96E-10	2.2437	2.2437
ST3GAL4	1.91E-08	2.2181	2.2181
NLN	4.50E-10	2.2173	2.2173
CKS2	6.93E-07	2.2061	2.2061
KIF11	5.04E-08	2.2018	2.2018
LINC01296	2.73E-09	2.1996	2.1996
LGR6	4.58E-04	2.1896	2.1896
MFAP2	6.36E-06	2.1787	2.1787
ADGRV1	9.71E-04	2.1508	2.1508
ENC1	1.67E-08	2.1483	2.1483
PDE9A	8.22E-07	2.1451	2.1451

B3GALT5	2.06E-04	2.1438	2.1438
MLC1	2.43E-06	2.1296	2.1296
PEG3	4.76E-07	2.1281	2.1281
GINS1	2.63E-09	2.1158	2.1158
CENPU	1.16E-08	2.1062	2.1062
CRABP2	3.01E-04	2.1042	2.1042
DSC3	4.26E-05	2.0959	2.0959
AARD	9.82E-06	2.0952	2.0952
ZFHX4	3.11E-04	2.0935	2.0935
ZNF726	8.05E-09	2.0879	2.0879
H19///RPS12	7.27E-04	2.0758	2.0758
CDK1	3.48E-07	2.0692	2.0692
TMEFF1	1.60E-08	2.0674	2.0674
FAM227A	3.39E-08	2.0663	2.0663
BUB1B	3.97E-08	2.0609	2.0609
DAPK1	6.35E-08	2.0482	2.0482
SERPINB5	1.85E-06	2.0396	2.0396
TRPS1	4.30E-06	2.0297	2.0297
FAT1	9.24E-13	2.0291	2.0291
APBA2	9.18E-08	2.0289	2.0289
TUSC3	1.90E-09	2.0288	2.0288
ZNF711	3.84E-10	2.0287	2.0287
SHCBP1	6.96E-06	2.0180	2.0180
MTHFD2	2.20E-09	2.0142	2.0142
SOX4	1.80E-11	2.0114	2.0114
DNAH14	1.29E-07	2.0093	2.0093
CDC42EP3	2.91E-07	2.0051	2.0051
CDCA7	4.98E-08	1.9951	1.9951
POLE2	1.73E-08	1.9875	1.9875
GCNT2	2.59E-06	1.9744	1.9744
HIST1H2BF	1.99E-05	1.9657	1.9657
ZNF738	2.54E-10	1.9527	1.9527
STK26	1.17E-11	1.9498	1.9498
CLUL1	1.63E-07	1.9350	1.9350
SLC35F3	2.15E-07	1.9308	1.9308
TMSB15B	4.79E-07	1.9283	1.9283
MKI67	4.87E-07	1.9281	1.9281
PGM2L1	1.17E-10	1.9252	1.9252
KIF23	3.04E-09	1.9179	1.9179
BCL2	5.30E-09	1.9177	1.9177
MBOAT1	1.22E-06	1.9174	1.9174
PLCH1	1.20E-04	1.9173	1.9173
FANCI	2.03E-07	1.9096	1.9096

PTPRT	8.49E-04	1.9065	1.9065
BGN	3.53E-06	1.9061	1.9061
FZD7	1.81E-10	1.9022	1.9022
SLC12A1	4.49E-04	1.9011	1.9011
PGAP1	7.52E-07	1.8985	1.8985
FLJ36840	5.59E-09	1.8946	1.8946
KIAA0101	1.32E-06	1.8916	1.8916
WEE1	3.85E-06	1.8902	1.8902
FAM111B	1.90E-06	1.8830	1.8830
TPX2	2.14E-07	1.8827	1.8827
FAM83B	3.85E-09	1.8738	1.8738
UBE2T	1.03E-07	1.8630	1.8630
PLK4	4.04E-08	1.8622	1.8622
IL17RB	2.12E-04	1.8609	1.8609
TAS2R4	1.60E-07	1.8574	1.8574
MELK	7.70E-08	1.8571	1.8571
NOTCH1	8.59E-08	1.8539	1.8539
PLCL1	3.18E-06	1.8528	1.8528
IGFBP2	4.40E-05	1.8464	1.8464
FAM178B	8.60E-06	1.8450	1.8450
CCDC34	6.31E-06	1.8429	1.8429
ZNF682	8.99E-09	1.8391	1.8391
TBC1D32	7.33E-09	1.8382	1.8382
AGPAT5	5.81E-08	1.8358	1.8358
OLFM2	6.82E-07	1.8319	1.8319
SNORD25	2.85E-06	1.8248	1.8248
COL27A1	1.45E-08	1.8212	1.8212
KNL1	4.27E-07	1.8202	1.8202
KDELC1	9.60E-09	1.8188	1.8188
MTHFD1L	7.49E-08	1.8147	1.8147
TYMS	2.19E-08	1.8120	1.8120
CENPI	1.17E-07	1.8115	1.8115
FADS2	1.01E-06	1.8082	1.8082
EN1	9.54E-11	1.7988	1.7988
SNORD28	2.23E-06	1.7962	1.7962
BUB1	4.13E-08	1.7923	1.7923
SNORD13P2	1.02E-09	1.7895	1.7895
TTK	4.44E-06	1.7892	1.7892
PITPNM2	1.52E-07	1.7786	1.7786
USP32P1	2.73E-05	1.7743	1.7743
RPGRIP1L	5.82E-09	1.7737	1.7737
NTRK3	3.26E-04	1.7707	1.7707
NHSL1	8.84E-08	1.7613	1.7613

WDHD1	1.61E-08	1.7561	1.7561
CCDC144A	8.63E-07	1.7548	1.7548
PSD3	1.59E-07	1.7541	1.7541
C21orf88	3.11E-04	1.7495	1.7495
DLGAP5	1.27E-06	1.7482	1.7482
IGSF3	3.33E-07	1.7476	1.7476
RASA1	3.37E-09	1.7414	1.7414
MCM7	1.42E-09	1.7376	1.7376
SCHIP1	9.71E-08	1.7317	1.7317
C4orf46	8.49E-10	1.7305	1.7305
GPX8	2.62E-05	1.7294	1.7294
SKA3	1.31E-06	1.7293	1.7293
PCDHB10	3.30E-05	1.7198	1.7198
MIA	2.37E-05	1.7154	1.7154
PLEKHG4B	3.63E-10	1.6943	1.6943
COL4A2	3.92E-05	1.6863	1.6863
EFNA3	2.87E-07	1.6853	1.6853
NCAPG	2.89E-07	1.6818	1.6818
HIST1H3B	2.10E-05	1.6780	1.6780
DGKH	7.78E-08	1.6777	1.6777
KPNA2	7.95E-08	1.6760	1.6760
DIAPH3	5.92E-08	1.6728	1.6728
ZWILCH	1.21E-09	1.6608	1.6608
SMOC2	2.62E-04	1.6402	1.6402
KIF20A	1.01E-07	1.6312	1.6312
ARHGAP11A	1.18E-06	1.6310	1.6310
RCN2	7.07E-09	1.6244	1.6244
SNORD27	3.66E-06	1.6231	1.6231
ATF7IP2	1.29E-04	1.6211	1.6211
WNK3	3.11E-06	1.6189	1.6189
SLC24A3	1.11E-08	1.6165	1.6165
MND1	1.98E-07	1.6149	1.6149
ARHGAP11B	2.41E-07	1.6127	1.6127
TP53	5.88E-09	1.6072	1.6072
TTLL4	1.08E-08	1.6054	1.6054
HIST1H4K	3.97E-07	1.5974	1.5974
PCDHB14	2.61E-05	1.5973	1.5973
TFAP2C	5.58E-08	1.5954	1.5954
CDH11	5.66E-04	1.5948	1.5948
CHRNA5	6.84E-09	1.5924	1.5924
SNORD4B	8.31E-07	1.5921	1.5921
LAMC2	1.01E-04	1.5890	1.5890
HIST1H1B	1.23E-05	1.5874	1.5874

CENPJ	1.78E-09	1.5873	1.5873
KIF14	1.68E-06	1.5868	1.5868
WDFY2	1.58E-10	1.5851	1.5851
BICD1	7.35E-07	1.5847	1.5847
SNORD26	5.55E-06	1.5800	1.5800
H2AFY2	4.57E-09	1.5797	1.5797
NUF2	6.17E-07	1.5786	1.5786
ASPM	4.27E-07	1.5772	1.5772
FBXL2	6.66E-09	1.5766	1.5766
MFAP3L	4.22E-05	1.5762	1.5762
KNTC1	1.24E-07	1.5760	1.5760
FGFR1	6.89E-06	1.5747	1.5747
ZNF675	6.96E-09	1.5729	1.5729
ALS2CR11	2.70E-06	1.5707	1.5707
ESCO2	7.77E-06	1.5692	1.5692
KIF15	4.13E-06	1.5683	1.5683
PRELP	1.08E-04	1.5680	1.5680
RAD51AP1	9.13E-07	1.5631	1.5631
COL4A1	6.78E-05	1.5596	1.5596
SGO1	3.61E-05	1.5570	1.5570
CEP170	2.22E-09	1.5540	1.5540
SLC2A1	7.19E-05	1.5526	1.5526
PBK	2.15E-05	1.5525	1.5525
LEF1	1.50E-04	1.5506	1.5506
ST6GALNAC5	1.03E-04	1.5490	1.5490
NUDT11	3.95E-08	1.5454	1.5454
GPSM2	4.05E-04	1.5447	1.5447
DSP	8.24E-10	1.5447	1.5447
ATAD5	4.53E-07	1.5444	1.5444
CCND1	7.14E-08	1.5382	1.5382
ATAT1	6.77E-09	1.5338	1.5338
ABCG1	1.00E-05	1.5251	1.5251
SNORD77	2.83E-07	1.5187	1.5187
SUGCT	6.34E-09	1.5168	1.5168
SNORD31	3.10E-06	1.5160	1.5160
TIAM1	1.70E-05	1.5151	1.5151
GGH	1.99E-08	1.5151	1.5151
ST8SIA1	4.90E-06	1.5148	1.5148
FAM198A	2.15E-05	1.5120	1.5120
ZNF681	8.60E-06	1.5113	1.5113
SPSB1	6.59E-07	1.5102	1.5102
HIST1H3I	3.09E-04	1.5098	1.5098
PARPBP	3.01E-07	1.5011	1.5011

CCNA2	9.02E-07	1.4980	1.4980
ZNF724	3.25E-06	1.4963	1.4963
SNORD52	2.20E-09	1.4933	1.4933
SPARC	5.96E-04	1.4932	1.4932
ANXA8	1.75E-05	1.4901	1.4901
DDIT4	1.83E-05	1.4890	1.4890
USP6	1.93E-07	1.4855	1.4855
CEP55	3.51E-07	1.4837	1.4837
NCKAP5	8.07E-04	1.4805	1.4805
GNB4	2.29E-05	1.4803	1.4803
C5orf34	9.67E-06	1.4789	1.4789
C14orf37	4.01E-07	1.4746	1.4746
SDK2	1.01E-05	1.4685	1.4685
C21orf91	5.49E-06	1.4677	1.4677
CCDC14	1.07E-08	1.4664	1.4664
APBB2	1.04E-05	1.4663	1.4663
PUS7	2.11E-08	1.4634	1.4634
ANKRD36	1.16E-08	1.4597	1.4597
MXRA5	3.07E-04	1.4589	1.4589
EPHX4	3.00E-06	1.4581	1.4581
EPHB3	7.80E-07	1.4560	1.4560
TESMIN	3.34E-09	1.4533	1.4533
DPY19L2	4.19E-06	1.4527	1.4527
ACSL3	1.56E-04	1.4527	1.4527
SVIL	1.28E-09	1.4525	1.4525
FAM69C	8.77E-05	1.4503	1.4503
TICRR	2.42E-06	1.4450	1.4450
HIST1H4L	2.98E-06	1.4357	1.4357
RTKN2	4.92E-06	1.4339	1.4339
ZNF260	7.98E-07	1.4297	1.4297
ANKRD50	1.36E-08	1.4285	1.4285
MRC2	1.52E-05	1.4267	1.4267
BCL2L11	8.56E-07	1.4258	1.4258
SLC45A4	4.45E-04	1.4255	1.4255
CHEK1	1.67E-05	1.4255	1.4255
PRKX	8.10E-08	1.4253	1.4253
KIT	7.86E-04	1.4236	1.4236
ZNF610	2.12E-04	1.4187	1.4187
HIST1H1A	3.32E-04	1.4185	1.4185
KIF18A	2.97E-06	1.4138	1.4138
GLMN	1.37E-08	1.4136	1.4136
KIAA1524	1.65E-07	1.4114	1.4114
SPAST	1.58E-08	1.4102	1.4102

TMEM97	2.91E-06	1.4093	1.4093
FAM101A	1.55E-04	1.4078	1.4078
CEP85	2.35E-08	1.4055	1.4055
KLF12	8.18E-07	1.4021	1.4021
NAPEPLD	3.34E-09	1.3989	1.3989
MYEF2	7.60E-05	1.3980	1.3980
ANTXR1	2.85E-04	1.3962	1.3962
TAS2R3	9.13E-07	1.3956	1.3956
BRCA2	4.55E-07	1.3939	1.3939
OR2L13	1.45E-04	1.3893	1.3893
PCDHB18P	4.10E-05	1.3888	1.3888
SERPINH1	2.27E-05	1.3862	1.3862
FLVCR1	3.30E-08	1.3791	1.3791
CENPE	2.71E-06	1.3785	1.3785
ZNF793	1.67E-06	1.3732	1.3732
CDC25B	2.85E-05	1.3729	1.3729
ZNF431	9.35E-09	1.3729	1.3729
RNF168	5.10E-04	1.3699	1.3699
HIST1H3F	9.94E-04	1.3696	1.3696
KCTD1	2.81E-07	1.3695	1.3695
LOX	7.32E-04	1.3692	1.3692
HIST1H2AE	8.87E-06	1.3683	1.3683
RAD54B	2.95E-07	1.3644	1.3644
LIN9	5.85E-06	1.3633	1.3633
FAM106A	3.93E-06	1.3632	1.3632
MICAL3	1.18E-06	1.3625	1.3625
E2F5	2.92E-06	1.3622	1.3622
SLC46A3	7.28E-04	1.3613	1.3613
TAF1D	1.40E-04	1.3604	1.3604
LRRCC1	1.58E-05	1.3604	1.3604
AFAP1	7.53E-10	1.3602	1.3602
LDLRAD4	3.91E-09	1.3584	1.3584
WDR12	1.86E-08	1.3572	1.3572
ARNT2	2.23E-07	1.3554	1.3554
GINS2	1.38E-05	1.3548	1.3548
PRC1	8.33E-07	1.3527	1.3527
MID1	1.27E-06	1.3496	1.3496
YEATS2	2.39E-10	1.3478	1.3478
ZNF43	1.77E-06	1.3466	1.3466
EPHA4	2.35E-04	1.3434	1.3434
MAP9	7.59E-09	1.3432	1.3432
KDM5B	4.78E-10	1.3423	1.3423
ZNF254	2.99E-07	1.3422	1.3422

C18orf54	7.99E-07	1.3397	1.3397
RPP40	1.38E-06	1.3379	1.3379
USP17L2	2.45E-06	1.3370	1.3370
P4HA1	6.05E-05	1.3329	1.3329
OR11H1	5.65E-04	1.3310	1.3310
CKAP2	8.99E-07	1.3299	1.3299
CALD1	9.04E-07	1.3288	1.3288
DPY19L2P2	9.77E-07	1.3276	1.3276
MPDZ	9.99E-06	1.3251	1.3251
NEIL3	8.18E-07	1.3247	1.3247
SH3PXD2B	1.36E-08	1.3204	1.3204
PLCG1	6.16E-10	1.3197	1.3197
RBL1	8.26E-08	1.3181	1.3181
TFRC	2.40E-08	1.3175	1.3175
E2F7	8.86E-06	1.3162	1.3162
IL17RD	5.48E-05	1.3127	1.3127
TAS2R10	5.21E-05	1.3078	1.3078
ZNF93	6.66E-07	1.3057	1.3057
SPAG5	5.98E-08	1.3029	1.3029
CHML	6.42E-10	1.2997	1.2997
SNHG15	2.08E-04	1.2992	1.2992
TIA1	1.62E-08	1.2980	1.2980
ZNF670	4.18E-07	1.2967	1.2967
EDA2R	3.04E-06	1.2938	1.2938
MCM4	3.45E-06	1.2924	1.2924
BICC1	1.07E-06	1.2909	1.2909
GPR137B	1.28E-07	1.2895	1.2895
WNK1	1.51E-06	1.2883	1.2883
ZNF135	1.22E-08	1.2872	1.2872
IGF1R	1.48E-08	1.2861	1.2861
PLXDC2	7.38E-08	1.2856	1.2856
MYO10	1.74E-06	1.2854	1.2854
ZNF124	1.21E-06	1.2852	1.2852
TM4SF1	3.49E-08	1.2838	1.2838
MTBP	2.74E-06	1.2828	1.2828
ARHGEF9	1.27E-06	1.2819	1.2819
EXO1	2.83E-06	1.2807	1.2807
TRIM45	1.19E-08	1.2802	1.2802
ZNF454	2.49E-07	1.2786	1.2786
PCNX1	1.01E-06	1.2781	1.2781
MYOF	3.28E-04	1.2775	1.2775
RUNX1	4.30E-06	1.2769	1.2769
PCSK6	5.63E-05	1.2756	1.2756

ZFP69B	1.78E-08	1.2730	1.2730
ZNF486	4.64E-06	1.2708	1.2708
CDC6	1.03E-05	1.2702	1.2702
PMS2P1	5.82E-08	1.2664	1.2664
SNORD22	3.26E-05	1.2661	1.2661
ETV5	1.49E-05	1.2658	1.2658
PTTG1	4.38E-07	1.2655	1.2655
NCS1	1.98E-04	1.2645	1.2645
SNORD79	6.25E-06	1.2636	1.2636
PCDHA10	9.27E-05	1.2634	1.2634
ZNF177	2.97E-06	1.2619	1.2619
SNORD54	1.89E-05	1.2617	1.2617
PCDHA6	7.35E-05	1.2616	1.2616
MAP4K4	3.24E-07	1.2608	1.2608
NEDD4	6.78E-04	1.2607	1.2607
SNORD78	1.21E-05	1.2597	1.2597
RASAL2	3.33E-06	1.2586	1.2586
PUM3	3.92E-06	1.2579	1.2579
EWSAT1	1.20E-05	1.2560	1.2560
ZNF713	1.33E-07	1.2556	1.2556
CACNB3	5.59E-07	1.2553	1.2553
ZFP1	2.07E-08	1.2546	1.2546
PKP1	4.15E-04	1.2531	1.2531
IP6K2	7.23E-09	1.2520	1.2520
BMP7	2.61E-04	1.2520	1.2520
TAS2R5	1.64E-06	1.2497	1.2497
CIT	8.12E-06	1.2475	1.2475
FAM60A	8.06E-06	1.2468	1.2468
ZC3HAV1L	3.75E-09	1.2465	1.2465
WDR35	4.58E-07	1.2446	1.2446
ABI2	5.50E-11	1.2443	1.2443
ZRANB3	6.57E-08	1.2416	1.2416
ZNF84	6.78E-09	1.2413	1.2413
FSCN1	9.60E-08	1.2400	1.2400
KALRN	1.89E-07	1.2378	1.2378
SCPEP1	2.86E-04	1.2357	1.2357
SLC39A10	2.28E-07	1.2337	1.2337
TCF7L1	7.33E-07	1.2329	1.2329
SPRED1	8.07E-06	1.2329	1.2329
ATP2C1	6.49E-05	1.2324	1.2324
AS3MT	9.51E-05	1.2313	1.2313
GLS	1.29E-06	1.2301	1.2301
CTPS2	5.62E-07	1.2296	1.2296

XKR9	4.17E-04	1.2275	1.2275
CDK19	1.78E-06	1.2266	1.2266
HOMER1	5.13E-06	1.2247	1.2247
PLXNB1	1.02E-08	1.2243	1.2243
GJC1	6.40E-04	1.2241	1.2241
RAD18	4.59E-08	1.2237	1.2237
GPX7	1.00E-04	1.2235	1.2235
RAP2A	2.04E-08	1.2230	1.2230
TBC1D1	5.99E-07	1.2229	1.2229
MCM3	5.37E-07	1.2227	1.2227
CDKN3	2.55E-05	1.2222	1.2222
USP32	3.64E-08	1.2202	1.2202
TUBA1A	2.87E-07	1.2197	1.2197
SNORD47	3.97E-07	1.2197	1.2197
YPEL1	3.87E-06	1.2160	1.2160
SPDL1	2.28E-07	1.2146	1.2146
PCDHB15	2.40E-04	1.2124	1.2124
DNAH2	2.05E-06	1.2121	1.2121
FZD3	8.85E-06	1.2099	1.2099
KLF7	1.36E-05	1.2092	1.2092
CDKL5	7.83E-06	1.2066	1.2066
TRIP13	3.64E-08	1.2057	1.2057
CEP290	7.49E-08	1.2057	1.2057
CTNNB1	1.23E-08	1.2042	1.2042
PAWR	7.77E-10	1.2035	1.2035
RRH	8.14E-04	1.2031	1.2031
PCNA	3.21E-07	1.2023	1.2023
ZNF850	2.70E-06	1.2021	1.2021
PARD6G	4.77E-06	1.2006	1.2006
HIST1H2AD	6.32E-05	1.1974	1.1974
KIF3A	1.02E-07	1.1954	1.1954
HIST2H2BA	3.04E-05	1.1938	1.1938
PSTPIP2	8.60E-06	1.1920	1.1920
WDSUB1	5.04E-08	1.1917	1.1917
ZNF649	9.59E-05	1.1903	1.1903
EPT1	2.04E-08	1.1884	1.1884
POGLUT1	7.12E-09	1.1876	1.1876
HNRNPA1L2	1.62E-09	1.1865	1.1865
ZNF83	2.06E-04	1.1847	1.1847
ZNF492	6.25E-06	1.1844	1.1844
KIAA1549L	2.04E-04	1.1830	1.1830
SPC25	5.65E-04	1.1811	1.1811
STX6	3.28E-10	1.1805	1.1805

B4GALNT3	6.67E-05	1.1786	1.1786
MEX3C	7.70E-11	1.1782	1.1782
CNOT6	1.14E-09	1.1776	1.1776
PALLD	7.00E-06	1.1747	1.1747
ZNF714	1.23E-07	1.1742	1.1742
ZNF519	5.36E-06	1.1733	1.1733
KLHL13	9.89E-04	1.1733	1.1733
BCL11A	5.61E-07	1.1722	1.1722
RUVBL1	3.78E-10	1.1716	1.1716
MAGED4B	2.36E-09	1.1709	1.1709
DLEU2	1.74E-04	1.1703	1.1703
ABCC5	2.04E-08	1.1699	1.1699
TM4SF18	4.73E-04	1.1697	1.1697
RCN3	4.53E-04	1.1694	1.1694
CENPH	2.51E-06	1.1687	1.1687
CCDC171	5.20E-06	1.1680	1.1680
MMS22L	1.77E-06	1.1675	1.1675
TRO	2.44E-06	1.1667	1.1667
ZNRD1ASP	1.60E-07	1.1646	1.1646
ZNF607	4.99E-06	1.1645	1.1645
FANCD2	3.45E-05	1.1628	1.1628
RPL7A	2.22E-06	1.1623	1.1623
CCDC88C	3.33E-05	1.1560	1.1560
MOK	7.19E-06	1.1542	1.1542
KRT17	6.07E-04	1.1528	1.1528
NUP93	7.09E-09	1.1520	1.1520
ZNF354A	9.91E-09	1.1513	1.1513
RPS3A	4.78E-06	1.1509	1.1509
PON2	3.18E-07	1.1503	1.1503
SRPK2	3.93E-07	1.1502	1.1502
KLRD1	5.41E-04	1.1494	1.1494
CDC7	2.46E-04	1.1489	1.1489
EXT1	8.54E-07	1.1450	1.1450
KATNBL1	2.45E-08	1.1445	1.1445
LY75	7.78E-04	1.1441	1.1441
EFNA4	1.02E-06	1.1441	1.1441
SNORD49A	4.60E-05	1.1427	1.1427
RACGAP1	3.93E-05	1.1419	1.1419
HENMT1	5.69E-08	1.1396	1.1396
RIBC2	1.35E-05	1.1368	1.1368
TRAM1L1	3.74E-08	1.1362	1.1362
HIST2H3C	4.14E-06	1.1362	1.1362
DDIAS	8.71E-05	1.1362	1.1362

SNORD1A	1.14E-05	1.1362	1.1362
NEK2	4.92E-06	1.1357	1.1357
CTTNBP2NL	1.28E-06	1.1356	1.1356
SQLE	2.94E-06	1.1353	1.1353
SNORA73A	1.45E-05	1.1328	1.1328
IFT80	2.04E-08	1.1327	1.1327
CHST3	1.79E-06	1.1308	1.1308
DCAF4	7.32E-06	1.1304	1.1304
TIMM8A	3.24E-08	1.1291	1.1291
OSBPL3	4.84E-05	1.1290	1.1290
FKBP7	1.05E-05	1.1279	1.1279
ZNF732	3.36E-06	1.1278	1.1278
ZNF512	4.10E-11	1.1271	1.1271
SLFN12	2.63E-06	1.1266	1.1266
CABYR	1.35E-06	1.1266	1.1266
CCNB1	7.83E-07	1.1255	1.1255
ISYNA1	2.14E-06	1.1245	1.1245
POLQ	1.28E-05	1.1233	1.1233
MET	2.90E-05	1.1204	1.1204
PDZRN3	5.95E-06	1.1201	1.1201
SKP2	1.31E-08	1.1199	1.1199
ZNF85	8.54E-08	1.1198	1.1198
FLNA	9.13E-05	1.1198	1.1198
ADARB1	2.58E-04	1.1197	1.1197
CLSPN	5.29E-05	1.1181	1.1181
CC2D2A	4.77E-05	1.1175	1.1175
ZNF695	1.66E-05	1.1174	1.1174
LUC7L	5.95E-10	1.1163	1.1163
NCAPG2	3.05E-07	1.1128	1.1128
KSR1	2.24E-05	1.1124	1.1124
CCDC8	2.50E-04	1.1112	1.1112
SNORD15A	2.29E-05	1.1099	1.1099
ZFAS1	3.23E-06	1.1096	1.1096
RCAN1	3.49E-04	1.1074	1.1074
CYTH2	9.51E-05	1.1072	1.1072
RBFOX2	4.73E-06	1.1062	1.1062
CERS6	2.15E-05	1.1039	1.1039
OR9A2	1.75E-05	1.0989	1.0989
ZNF131	1.97E-07	1.0987	1.0987
SNORD4A	6.48E-06	1.0983	1.0983
CEP95	4.05E-11	1.0976	1.0976
TRIM37	7.07E-09	1.0975	1.0975
GPR137C	1.50E-05	1.0968	1.0968

PHF14	8.07E-08	1.0963	1.0963
SGO2	5.12E-06	1.0952	1.0952
HERC2	2.08E-06	1.0948	1.0948
ZNF667	5.81E-08	1.0947	1.0947
TBC1D7	7.04E-05	1.0938	1.0938
GUCY1B3	7.88E-05	1.0922	1.0922
RNF212	2.77E-04	1.0921	1.0921
PLEKHG1	5.15E-04	1.0908	1.0908
ZNF876P	7.21E-04	1.0905	1.0905
SLC9B2	1.52E-05	1.0888	1.0888
ZNF90	1.85E-08	1.0884	1.0884
TMEM98	4.66E-07	1.0863	1.0863
CLUAP1	1.06E-06	1.0842	1.0842
RFC3	9.55E-06	1.0841	1.0841
ZNF121	3.34E-09	1.0841	1.0841
HSPH1	2.66E-04	1.0836	1.0836
CCDC113	4.52E-05	1.0833	1.0833
GABBR1	8.79E-08	1.0830	1.0830
CDCA2	3.49E-04	1.0804	1.0804
CDC45	9.10E-06	1.0800	1.0800
SMC4	8.51E-07	1.0778	1.0778
KIF22	2.57E-09	1.0777	1.0777
MCM6	1.46E-05	1.0775	1.0775
PKI55	3.19E-07	1.0768	1.0768
DNMT1	8.79E-08	1.0766	1.0766
BZW2	1.85E-06	1.0748	1.0748
SNORD44	4.85E-07	1.0747	1.0747
HMGB3	1.01E-08	1.0744	1.0744
MGME1	1.29E-06	1.0736	1.0736
POLH	3.30E-05	1.0735	1.0735
LAPTM4B	4.14E-07	1.0732	1.0732
CHD1L	6.52E-08	1.0718	1.0718
PCDHA11	8.32E-05	1.0717	1.0717
PSPC1	5.50E-11	1.0717	1.0717
KCNG1	1.57E-04	1.0712	1.0712
CEP83	6.12E-09	1.0702	1.0702
PRR11	1.10E-06	1.0690	1.0690
ZNF544	7.20E-08	1.0684	1.0684
ZNF529	4.47E-06	1.0683	1.0683
APEX1	6.69E-09	1.0682	1.0682
ANP32E	1.78E-05	1.0668	1.0668
CLHC1	3.10E-06	1.0657	1.0657
ERRFI1	1.89E-05	1.0657	1.0657

ANKRD36B	1.49E-06	1.0653	1.0653
GEN1	5.51E-04	1.0653	1.0653
FIGNL1	4.09E-06	1.0652	1.0652
ARHGEF2	2.64E-06	1.0649	1.0649
DEPDC1	6.47E-04	1.0647	1.0647
NOTCH3	4.08E-06	1.0638	1.0638
DDX58	2.75E-05	1.0635	1.0635
SNORD75	3.85E-04	1.0629	1.0629
SNORD30	9.85E-04	1.0627	1.0627
TFAP2A	3.83E-05	1.0616	1.0616
ZNF257	3.54E-05	1.0612	1.0612
GPC4	2.00E-04	1.0598	1.0598
KIF20B	1.43E-05	1.0593	1.0593
FBXO22	3.45E-09	1.0590	1.0590
MCM10	5.09E-05	1.0588	1.0588
PLK1	4.51E-06	1.0558	1.0558
PCGF3	5.35E-07	1.0556	1.0556
FRAS1	7.17E-04	1.0552	1.0552
AJUBA	3.02E-05	1.0547	1.0547
TMEM237	2.17E-04	1.0540	1.0540
FOXM1	4.96E-06	1.0535	1.0535
APP	1.66E-06	1.0534	1.0534
ZNF749	5.95E-06	1.0533	1.0533
HDGFRP3	6.69E-07	1.0530	1.0530
ZNF107	1.11E-06	1.0528	1.0528
MGC70870	2.03E-04	1.0518	1.0518
DKC1	9.10E-07	1.0516	1.0516
HIST2H2BE	1.47E-05	1.0471	1.0471
KDM3A	4.90E-09	1.0462	1.0462
TBL1X	6.85E-07	1.0461	1.0461
HS6ST1	6.89E-08	1.0456	1.0456
STK36	3.45E-07	1.0454	1.0454
XPO1	1.67E-09	1.0446	1.0446
PNMAL1	4.77E-05	1.0443	1.0443
CA13	6.83E-05	1.0438	1.0438
KHDRBS1	7.69E-11	1.0427	1.0427
HN1	1.09E-05	1.0418	1.0418
HMGB2	3.15E-05	1.0413	1.0413
RFX3	6.92E-04	1.0392	1.0392
ZNF627	1.36E-07	1.0392	1.0392
ZFP82	7.02E-05	1.0391	1.0391
ZGRF1	8.15E-05	1.0383	1.0383
ZNF585B	1.09E-04	1.0383	1.0383

TRIM59	1.34E-05	1.0381	1.0381
GAS5	3.26E-05	1.0379	1.0379
CCT6A	2.11E-07	1.0373	1.0373
C1orf112	2.64E-06	1.0373	1.0373
ZFP37	1.14E-06	1.0322	1.0322
SMARCC1	1.65E-07	1.0320	1.0320
STK38	1.08E-07	1.0290	1.0290
TIGD1	3.61E-09	1.0288	1.0288
PTK7	8.49E-07	1.0284	1.0284
MTF2	9.39E-07	1.0283	1.0283
CCDC138	2.82E-06	1.0269	1.0269
POLR1A	3.90E-08	1.0247	1.0247
IGSF9	2.78E-08	1.0245	1.0245
CHD6	3.04E-09	1.0239	1.0239
PRNP	6.67E-04	1.0233	1.0233
TEX10	3.91E-09	1.0231	1.0231
CPSF6	3.83E-09	1.0229	1.0229
HNRNPR	1.60E-08	1.0181	1.0181
INTS7	1.64E-05	1.0181	1.0181
ZNF660	3.19E-06	1.0169	1.0169
MPHOSPH9	4.04E-08	1.0158	1.0158
TRIO	9.99E-07	1.0158	1.0158
ELOVL6	9.43E-05	1.0147	1.0147
ZNF146	7.50E-07	1.0135	1.0135
FLJ35816	6.21E-06	1.0133	1.0133
KIF3C	7.75E-09	1.0129	1.0129
ZNF320	1.63E-04	1.0121	1.0121
ZBED4	8.88E-10	1.0118	1.0118
TEAD2	2.81E-06	1.0100	1.0100
WDR27	1.49E-04	1.0092	1.0092
KLRA1P	3.15E-05	1.0091	1.0091
DOCK7	7.23E-07	1.0083	1.0083
CCDC82	3.29E-04	1.0079	1.0079
SNORD61	2.38E-04	1.0078	1.0078
VGLL4	4.02E-07	1.0060	1.0060
MAGOHB	3.32E-07	1.0050	1.0050
TAS2R13	2.14E-05	1.0049	1.0049
PGM2	6.91E-05	1.0044	1.0044
WDPCP	4.11E-06	1.0036	1.0036
RNF165	1.74E-05	1.0034	1.0034
KDM1A	1.16E-07	1.0031	1.0031
GINS4	3.52E-05	1.0023	1.0023
OTUD3	9.04E-07	1.0006	1.0006

HACE1	1.11E-05	1.0002	1.0002
IL13RA1	7.65E-05	1.0001	1.0001

B, Downregulated

Gene ID	FDR	Log2 FC	|Log2 FC|
COLGALT2	5.54E-05	-1.0011	1.0011
SHISA4	4.53E-07	-1.0018	1.0018
ERO1A	2.78E-04	-1.0031	1.0031
ME3	1.51E-08	-1.0038	1.0038
ACVRL1	6.28E-07	-1.0041	1.0041
SLC39A2	1.21E-04	-1.0054	1.0054
TAB2	2.80E-05	-1.0063	1.0063
CAT	1.22E-05	-1.0071	1.0071
MAPK13	2.10E-05	-1.0085	1.0085
NRXN1	2.95E-06	-1.0088	1.0088
HLF	5.72E-05	-1.0097	1.0097
RAB3D	3.14E-06	-1.0098	1.0098
ADAMTSL1	1.27E-04	-1.0116	1.0116
KIF5B	1.98E-04	-1.0134	1.0134
CLVS1	4.79E-07	-1.0137	1.0137
HSPB7	1.33E-04	-1.0150	1.0150
EPB41L1	4.38E-06	-1.0187	1.0187
LRG1	1.64E-07	-1.0194	1.0194
ST6GALNAC6	1.07E-08	-1.0202	1.0202
DOPEY2	1.64E-05	-1.0207	1.0207
UAP1	5.95E-07	-1.0217	1.0217
SP100	3.43E-05	-1.0217	1.0217
DHRS2	1.32E-05	-1.0221	1.0221
ARFGAP3	8.35E-07	-1.0227	1.0227
HPN-AS1	2.33E-05	-1.0245	1.0245
PDHA1	2.12E-08	-1.0258	1.0258
SCN9A	6.86E-07	-1.0258	1.0258
FZD5	7.87E-07	-1.0264	1.0264
SYT7	3.69E-04	-1.0265	1.0265
COX14	9.29E-06	-1.0269	1.0269
SPRY2	3.38E-04	-1.0283	1.0283
ADGRB3	1.84E-05	-1.0297	1.0297
TCIRG1	8.62E-07	-1.0309	1.0309
GRAMD1A	3.45E-05	-1.0330	1.0330
FOXQ1	5.49E-06	-1.0345	1.0345
TMEM61	7.45E-07	-1.0349	1.0349
GCH1	4.59E-04	-1.0353	1.0353
CALM1	2.28E-07	-1.0355	1.0355
TMEM132B	1.70E-07	-1.0361	1.0361

PCYOX1	4.83E-06	-1.0413	1.0413
DNAJC1	1.28E-06	-1.0414	1.0414
STARD10	1.92E-05	-1.0423	1.0423
IDNK	6.78E-09	-1.0424	1.0424
PKP2	3.83E-05	-1.0439	1.0439
GALNT15	8.56E-05	-1.0455	1.0455
BMP3	2.59E-08	-1.0483	1.0483
TSPAN6	2.61E-06	-1.0486	1.0486
TMEM181	5.26E-06	-1.0490	1.0490
BMP5	1.36E-06	-1.0495	1.0495
FBXW10	3.84E-05	-1.0507	1.0507
C1orf87	5.36E-06	-1.0510	1.0510
TNXA	1.24E-04	-1.0510	1.0510
NFIA	1.84E-04	-1.0527	1.0527
TMEM62	5.77E-08	-1.0555	1.0555
GAL3ST4	4.93E-04	-1.0561	1.0561
MGST2	6.40E-06	-1.0562	1.0562
PLCB1	5.37E-05	-1.0588	1.0588
GABARAPL1	5.28E-07	-1.0597	1.0597
CSN2	1.36E-07	-1.0604	1.0604
ALS2CR12	3.20E-07	-1.0618	1.0618
TRHDE	7.06E-06	-1.0628	1.0628
PLP1	4.76E-04	-1.0628	1.0628
SYVN1	3.49E-08	-1.0657	1.0657
FAM63A	1.05E-06	-1.0662	1.0662
KIAA0513	1.35E-07	-1.0692	1.0692
STXBP1	4.00E-07	-1.0717	1.0717
PPP2R5A	1.58E-06	-1.0719	1.0719
DCXR	1.46E-05	-1.0746	1.0746
VAMP8	3.21E-06	-1.0754	1.0754
FKBP11	2.23E-06	-1.0764	1.0764
CNTFR	5.25E-04	-1.0765	1.0765
FAXDC2	2.52E-05	-1.0777	1.0777
CES4A	6.72E-06	-1.0787	1.0787
TGFBR2	1.19E-04	-1.0789	1.0789
ECHS1	8.29E-11	-1.0805	1.0805
C2orf88	5.28E-09	-1.0807	1.0807
CTSL	6.98E-06	-1.0809	1.0809
TNS2	9.58E-07	-1.0814	1.0814
SOBP	2.01E-04	-1.0838	1.0838
CYP4B1	4.25E-04	-1.0850	1.0850
MTUS1	6.72E-05	-1.0851	1.0851
PDK2	8.10E-08	-1.0857	1.0857

LRRC2	2.47E-07	-1.0866	1.0866
TTC7B	7.10E-08	-1.0889	1.0889
PMP2	1.11E-06	-1.0901	1.0901
HERPUD1	1.49E-05	-1.0928	1.0928
IFI35	9.79E-09	-1.0929	1.0929
RRBP1	3.75E-09	-1.0939	1.0939
AGTR1	3.00E-05	-1.0949	1.0949
SEC23B	1.10E-07	-1.0958	1.0958
SLC2A4	4.89E-09	-1.0983	1.0983
CBX7	3.82E-07	-1.1000	1.1000
RGN	5.27E-05	-1.1014	1.1014
SCAMP2	3.01E-07	-1.1030	1.1030
KBTBD12	9.24E-08	-1.1047	1.1047
SLC50A1	6.12E-07	-1.1055	1.1055
RAB15	1.22E-04	-1.1074	1.1074
SIK2	8.33E-06	-1.1102	1.1102
DPAGT1	4.09E-07	-1.1107	1.1107
MAPT	6.01E-06	-1.1112	1.1112
CNR1	3.58E-05	-1.1143	1.1143
P4HB	3.21E-05	-1.1146	1.1146
GNMT	1.93E-07	-1.1185	1.1185
RAPGEF3	1.67E-09	-1.1196	1.1196
HEPACAM2	6.01E-07	-1.1196	1.1196
PDZD8	7.53E-09	-1.1216	1.1216
BMP4	1.13E-05	-1.1225	1.1225
LGALS12	1.90E-04	-1.1246	1.1246
SRXN1	1.49E-05	-1.1248	1.1248
PTGER3	3.52E-06	-1.1252	1.1252
BACE2	3.19E-07	-1.1253	1.1253
TMEM109	7.12E-07	-1.1277	1.1277
CTH	5.22E-04	-1.1306	1.1306
TRAK2	2.51E-09	-1.1311	1.1311
PITX1	2.35E-06	-1.1332	1.1332
RETSAT	3.23E-06	-1.1367	1.1367
PODN	7.87E-04	-1.1385	1.1385
CEBPD	1.80E-04	-1.1418	1.1418
DNAH7	2.08E-06	-1.1421	1.1421
MAB21L3	2.61E-07	-1.1425	1.1425
ST3GAL1	6.11E-07	-1.1435	1.1435
CYP39A1	1.67E-04	-1.1442	1.1442
RNLS	2.14E-08	-1.1443	1.1443
MOCS2	1.92E-06	-1.1445	1.1445
KCNK6	5.61E-07	-1.1496	1.1496

CPTP	3.61E-06	-1.1502	1.1502
SEC61B	8.24E-10	-1.1518	1.1518
ERO1B	5.21E-09	-1.1537	1.1537
ADIPOR1	6.66E-09	-1.1558	1.1558
NRK	4.33E-07	-1.1564	1.1564
NKAIN3	2.08E-05	-1.1590	1.1590
PLPP3	4.58E-04	-1.1594	1.1594
ARL4A	6.80E-06	-1.1629	1.1629
KRT18	6.38E-04	-1.1629	1.1629
TUBA4A	2.02E-05	-1.1646	1.1646
UG0898H09	4.77E-07	-1.1677	1.1677
TSTA3	7.73E-07	-1.1696	1.1696
CCDC69	3.30E-04	-1.1713	1.1713
NAMPT	4.19E-04	-1.1725	1.1725
CIDEA	3.14E-05	-1.1740	1.1740
B3GALT1	8.54E-05	-1.1748	1.1748
LDLRAP1	1.35E-06	-1.1749	1.1749
GALE	9.69E-08	-1.1751	1.1751
ENPP4	3.06E-05	-1.1765	1.1765
ASS1	2.18E-04	-1.1769	1.1769
SLC40A1	1.48E-05	-1.1782	1.1782
MCTP2	2.49E-04	-1.1786	1.1786
KIAA2022	1.49E-08	-1.1807	1.1807
UBA7	5.19E-05	-1.1818	1.1818
UGT2B7	4.40E-04	-1.1843	1.1843
NOVA1	7.89E-06	-1.1856	1.1856
CRAT	1.39E-08	-1.1862	1.1862
C14orf128	2.05E-06	-1.1880	1.1880
NDRG2	2.02E-04	-1.1887	1.1887
ERN1	1.26E-06	-1.1890	1.1890
LDHD	8.13E-09	-1.1921	1.1921
PLSCR4	6.30E-04	-1.1969	1.1969
RAB17	4.51E-07	-1.2058	1.2058
GCOM1	7.91E-06	-1.2059	1.2059
IER3	8.94E-04	-1.2063	1.2063
STX19	2.54E-04	-1.2064	1.2064
IFIT1	2.24E-08	-1.2097	1.2097
NQO1	7.80E-05	-1.2145	1.2145
ATG4A	2.07E-09	-1.2148	1.2148
CHAD	8.06E-05	-1.2180	1.2180
SDCBP	3.42E-08	-1.2233	1.2233
ACOT11	3.89E-06	-1.2244	1.2244
TMEM30B	2.76E-05	-1.2268	1.2268

TST	8.87E-06	-1.2271	1.2271
ACOX2	3.73E-08	-1.2288	1.2288
ALDH3A2	1.64E-04	-1.2289	1.2289
KRT80	5.02E-06	-1.2301	1.2301
SPR	8.99E-08	-1.2310	1.2310
CYSTM1	6.80E-08	-1.2311	1.2311
LY9	1.84E-04	-1.2316	1.2316
AGPAT2	1.41E-07	-1.2321	1.2321
CARD6	2.69E-07	-1.2336	1.2336
NEBL	6.94E-04	-1.2339	1.2339
COL6A6	1.03E-07	-1.2357	1.2357
TFPI2	5.42E-08	-1.2366	1.2366
SLC15A2	3.61E-04	-1.2378	1.2378
HSDL2	4.13E-08	-1.2392	1.2392
TMBIM1	6.97E-05	-1.2409	1.2409
TNFSF10	3.33E-05	-1.2445	1.2445
SLC9A3R1	1.38E-05	-1.2464	1.2464
AACS	5.07E-06	-1.2474	1.2474
NBEA	4.14E-04	-1.2503	1.2503
NPY6R	2.50E-06	-1.2505	1.2505
TEC	2.04E-07	-1.2518	1.2518
CCL14	2.97E-04	-1.2551	1.2551
PRSS8	1.51E-04	-1.2558	1.2558
RAB26	1.76E-07	-1.2561	1.2561
WBP2	2.76E-06	-1.2568	1.2568
CD40	5.88E-08	-1.2574	1.2574
EPAS1	2.38E-05	-1.2599	1.2599
ABCC6	3.47E-07	-1.2599	1.2599
RILPL2	7.32E-09	-1.2606	1.2606
RIMS1	4.17E-07	-1.2607	1.2607
ANG	3.58E-06	-1.2610	1.2610
SP140L	1.40E-04	-1.2617	1.2617
EPHX1	3.33E-11	-1.2635	1.2635
SEC62	1.52E-11	-1.2651	1.2651
PDCD4	1.96E-07	-1.2653	1.2653
CRISP2	3.68E-04	-1.2657	1.2657
CD34	1.79E-04	-1.2689	1.2689
DNAJB9	9.23E-09	-1.2699	1.2699
HECTD3	9.72E-08	-1.2703	1.2703
LBH	2.49E-05	-1.2705	1.2705
ZNF704	4.54E-05	-1.2705	1.2705
RBM47	1.32E-05	-1.2710	1.2710
TMEM37	9.09E-07	-1.2740	1.2740

IL6ST	9.44E-06	-1.2748	1.2748
NCEH1	1.82E-05	-1.2762	1.2762
NMRK1	9.27E-05	-1.2796	1.2796
ZDHHC2	3.06E-06	-1.2807	1.2807
ZNF827	8.53E-07	-1.2826	1.2826
SIDT2	4.52E-08	-1.2828	1.2828
ADAMTSL3	2.37E-05	-1.2834	1.2834
SH3BP4	1.73E-05	-1.2890	1.2890
SKAP2	4.72E-05	-1.2892	1.2892
CES1	1.32E-04	-1.2898	1.2898
LDB2	1.53E-05	-1.2949	1.2949
AGMO	5.28E-04	-1.2980	1.2980
FICD	8.92E-09	-1.3014	1.3014
TBC1D24	2.54E-06	-1.3059	1.3059
CDH12	9.76E-05	-1.3069	1.3069
CASP4	1.65E-08	-1.3074	1.3074
CXXC5	1.65E-07	-1.3076	1.3076
RHOU	3.15E-06	-1.3089	1.3089
ALAS1	4.38E-07	-1.3093	1.3093
H6PD	7.30E-07	-1.3110	1.3110
DNAJC3	1.95E-09	-1.3117	1.3117
CRYL1	1.03E-07	-1.3152	1.3152
NAALADL2	5.75E-04	-1.3176	1.3176
FES	8.12E-08	-1.3177	1.3177
PITPNC1	8.56E-07	-1.3187	1.3187
DGKA	1.06E-05	-1.3189	1.3189
ARG2	4.07E-05	-1.3216	1.3216
CAMKK1	3.05E-08	-1.3277	1.3277
SRPRA	6.95E-08	-1.3299	1.3299
MYO1C	2.55E-07	-1.3319	1.3319
FOLR1	8.07E-04	-1.3325	1.3325
SLC5A7	3.23E-06	-1.3384	1.3384
CAMK2N1	5.52E-04	-1.3391	1.3391
MECOM	9.15E-06	-1.3396	1.3396
TMEM99	2.41E-05	-1.3398	1.3398
NOS1	8.83E-07	-1.3422	1.3422
AOX1	6.38E-06	-1.3455	1.3455
SULT1C2	6.57E-08	-1.3506	1.3506
ICAM3	3.28E-06	-1.3514	1.3514
CPED1	5.56E-04	-1.3576	1.3576
TSHZ2	6.92E-05	-1.3590	1.3590
CITED2	1.43E-05	-1.3601	1.3601
BNIP3	5.83E-07	-1.3601	1.3601

FRMD4B	8.21E-07	-1.3607	1.3607
NXF3	2.84E-05	-1.3627	1.3627
TLR6	1.86E-05	-1.3660	1.3660
INPP5J	1.68E-07	-1.3671	1.3671
EXPH5	5.71E-05	-1.3695	1.3695
FUT6	1.75E-08	-1.3699	1.3699
HOMER2	1.15E-04	-1.3713	1.3713
NR3C2	6.80E-07	-1.3714	1.3714
MARCH3	1.37E-05	-1.3719	1.3719
COL21A1	1.34E-06	-1.3724	1.3724
PC	2.57E-08	-1.3745	1.3745
SLC1A5	4.16E-04	-1.3746	1.3746
ARHGAP26	3.82E-07	-1.3746	1.3746
SCGB3A1	1.76E-04	-1.3748	1.3748
NUDT4	1.23E-05	-1.3805	1.3805
PPP1R36	4.73E-08	-1.3837	1.3837
BVES	2.54E-04	-1.3866	1.3866
SQRDL	5.59E-07	-1.3869	1.3869
REPS2	1.96E-06	-1.3873	1.3873
RASD1	4.07E-07	-1.3884	1.3884
CPEB4	3.44E-07	-1.3909	1.3909
TSPAN12	1.91E-05	-1.3940	1.3940
ENTPD3	4.45E-05	-1.3948	1.3948
CREB3L1	7.14E-05	-1.3956	1.3956
PCK1	3.71E-04	-1.3971	1.3971
SH2D4A	3.48E-08	-1.3994	1.3994
NANS	3.18E-07	-1.4002	1.4002
TSPAN5	7.13E-04	-1.4005	1.4005
INSIG1	8.40E-05	-1.4022	1.4022
ZCCHC2	2.50E-07	-1.4062	1.4062
HCAR1	1.63E-04	-1.4131	1.4131
PACS1	8.84E-08	-1.4135	1.4135
TNFSF15	4.84E-07	-1.4138	1.4138
GAREM1	1.18E-06	-1.4158	1.4158
NFATC2	1.65E-05	-1.4231	1.4231
VSIG10L	3.65E-06	-1.4232	1.4232
GLYAT	6.03E-04	-1.4244	1.4244
SERINC2	3.00E-05	-1.4248	1.4248
SRGAP1	6.79E-07	-1.4253	1.4253
PLPP5	1.68E-08	-1.4257	1.4257
ATP8A1	3.18E-07	-1.4271	1.4271
SV2B	1.35E-05	-1.4289	1.4289
VOPP1	1.62E-06	-1.4306	1.4306

SOD2	1.34E-04	-1.4308	1.4308
EIF4E3	7.31E-07	-1.4348	1.4348
PLIN2	4.98E-07	-1.4348	1.4348
SLC43A1	3.01E-09	-1.4354	1.4354
ACP2	9.69E-08	-1.4358	1.4358
GPR146	1.42E-07	-1.4360	1.4360
SLITRK5	1.83E-05	-1.4370	1.4370
SLC4A4	7.05E-06	-1.4375	1.4375
TXK	2.50E-04	-1.4401	1.4401
KCTD14	6.89E-05	-1.4409	1.4409
HSPB6	1.43E-04	-1.4454	1.4454
TMEM56	2.37E-04	-1.4486	1.4486
DENND3	1.13E-06	-1.4490	1.4490
KCNIP2	9.60E-04	-1.4492	1.4492
CAPN6	1.42E-04	-1.4502	1.4502
PELI2	1.07E-06	-1.4521	1.4521
FAM107B	1.34E-05	-1.4559	1.4559
ST3GAL6	9.78E-07	-1.4563	1.4563
GALNT13	5.40E-04	-1.4566	1.4566
SLC2A12	8.63E-05	-1.4580	1.4580
STXBP6	2.97E-09	-1.4601	1.4601
TMEM8A	1.36E-08	-1.4611	1.4611
NRG4	4.36E-06	-1.4617	1.4617
C4orf19	1.35E-05	-1.4633	1.4633
BCKDK	3.46E-07	-1.4665	1.4665
COLCA1	9.96E-05	-1.4667	1.4667
KREMEN1	9.92E-06	-1.4682	1.4682
SELENBP1	1.65E-04	-1.4691	1.4691
RAB27B	4.45E-06	-1.4742	1.4742
FLVCR2	9.22E-06	-1.4758	1.4758
SBSPON	3.35E-09	-1.4797	1.4797
DHDDS	1.95E-09	-1.4809	1.4809
SRPX	2.07E-05	-1.4836	1.4836
EMCN	3.57E-04	-1.4837	1.4837
GGT6	4.45E-07	-1.4841	1.4841
GRIA4	1.86E-10	-1.4859	1.4859
CLCNKB	4.48E-08	-1.4879	1.4879
SYBU	1.77E-07	-1.4882	1.4882
SLC17A5	3.75E-07	-1.4887	1.4887
LIPE	2.54E-04	-1.4897	1.4897
CDR1	4.01E-04	-1.4902	1.4902
GREM2	7.07E-09	-1.4928	1.4928
NUPR1	1.40E-07	-1.4940	1.4940

ARL4D	2.39E-08	-1.4944	1.4944
GPD1L	3.42E-07	-1.4945	1.4945
SCNN1A	2.96E-04	-1.4972	1.4972
HM13	8.25E-10	-1.5004	1.5004
LYPD1	3.49E-04	-1.5009	1.5009
FAM124A	9.30E-06	-1.5016	1.5016
PGR	5.09E-08	-1.5038	1.5038
HBEGF	1.92E-05	-1.5044	1.5044
SLC25A20	1.03E-09	-1.5050	1.5050
LYPD6B	9.90E-06	-1.5096	1.5096
CHST6	1.83E-06	-1.5112	1.5112
CD55	2.50E-07	-1.5133	1.5133
KDELR3	2.78E-06	-1.5137	1.5137
NIPAL2	6.73E-07	-1.5167	1.5167
PRKCA	7.06E-06	-1.5173	1.5173
COL28A1	4.64E-06	-1.5210	1.5210
OAS1	5.91E-09	-1.5218	1.5218
CCPG1	3.85E-09	-1.5220	1.5220
XDH	1.60E-05	-1.5262	1.5262
SH3RF2	1.37E-04	-1.5262	1.5262
MRAP2	6.59E-09	-1.5294	1.5294
BARX2	4.24E-04	-1.5336	1.5336
DLK1	2.89E-07	-1.5360	1.5360
CPLX3	1.45E-05	-1.5367	1.5367
CGNL1	6.31E-06	-1.5372	1.5372
FAM84B	1.60E-07	-1.5373	1.5373
ALAD	1.91E-08	-1.5380	1.5380
RASSF5	2.65E-05	-1.5402	1.5402
SPCS3	4.10E-11	-1.5412	1.5412
MLKL	8.26E-10	-1.5414	1.5414
RASEF	1.26E-05	-1.5425	1.5425
MEIS1	5.69E-04	-1.5481	1.5481
CEP85L	2.07E-07	-1.5499	1.5499
MBNL3	1.27E-06	-1.5502	1.5502
FKBP5	5.65E-04	-1.5544	1.5544
HLA-DMA	5.91E-05	-1.5546	1.5546
RND1	1.33E-05	-1.5550	1.5550
ALDH18A1	1.49E-08	-1.5558	1.5558
TMED3	1.65E-08	-1.5562	1.5562
CST3	2.41E-06	-1.5578	1.5578
S100A1	1.42E-07	-1.5638	1.5638
KLF15	9.12E-11	-1.5640	1.5640
TNS1	4.12E-05	-1.5673	1.5673

WFDC2	6.54E-05	-1.5677	1.5677
RHOBTB3	9.51E-07	-1.5700	1.5700
COL15A1	3.34E-05	-1.5700	1.5700
CTBS	1.02E-08	-1.5733	1.5733
LPAR1	2.51E-04	-1.5750	1.5750
TAC1	4.33E-06	-1.5753	1.5753
ARHGAP29	8.39E-07	-1.5754	1.5754
EIF4EBP1	3.28E-07	-1.5761	1.5761
FAM20C	2.89E-07	-1.5772	1.5772
HS3ST3B1	1.71E-08	-1.5783	1.5783
PIP5K1B	5.86E-04	-1.5828	1.5828
SMAD9	1.06E-07	-1.5846	1.5846
DAPL1	1.30E-06	-1.5858	1.5858
MCOLN3	4.10E-05	-1.5861	1.5861
PRKAA2	1.19E-05	-1.5874	1.5874
LPAR3	7.62E-06	-1.5902	1.5902
RORC	4.67E-06	-1.5903	1.5903
FRMPD1	1.73E-08	-1.5961	1.5961
RRAS	2.31E-08	-1.5987	1.5987
COBLL1	5.07E-05	-1.5989	1.5989
KIF21A	2.34E-06	-1.5997	1.5997
CTPS1	4.54E-07	-1.6014	1.6014
GGTA1P	3.57E-04	-1.6032	1.6032
SPX	1.94E-06	-1.6041	1.6041
UNC5C	4.07E-04	-1.6042	1.6042
ACSS2	1.60E-08	-1.6065	1.6065
TTLL7	1.24E-06	-1.6074	1.6074
IL1B	3.83E-05	-1.6088	1.6088
EPHA2	1.02E-07	-1.6156	1.6156
RSPO3	2.89E-07	-1.6158	1.6158
RNF148	3.49E-09	-1.6252	1.6252
ANO4	1.30E-06	-1.6253	1.6253
TSPAN33	3.69E-06	-1.6261	1.6261
AMPD1	4.69E-05	-1.6270	1.6270
MPP1	5.14E-07	-1.6284	1.6284
FMO5	1.86E-05	-1.6286	1.6286
NECAB1	1.61E-06	-1.6306	1.6306
CD14	9.07E-07	-1.6336	1.6336
CPEB3	2.68E-06	-1.6363	1.6363
LONRF3	3.82E-05	-1.6377	1.6377
SLC35F2	2.96E-06	-1.6416	1.6416
FZD4	2.71E-07	-1.6427	1.6427
LIMCH1	1.42E-05	-1.6446	1.6446

PDE11A	4.21E-06	-1.6467	1.6467
SELE	5.31E-04	-1.6467	1.6467
C17orf77	7.01E-06	-1.6502	1.6502
ABLIM1	1.06E-05	-1.6529	1.6529
TSPAN13	2.27E-07	-1.6604	1.6604
TSPAN15	3.70E-08	-1.6619	1.6619
AGMAT	1.05E-08	-1.6678	1.6678
MELTF	3.90E-08	-1.6708	1.6708
FAM46A	7.81E-07	-1.6749	1.6749
SLC16A7	1.16E-05	-1.6787	1.6787
XK	1.57E-08	-1.6793	1.6793
PDE7B	3.67E-06	-1.6813	1.6813
TNFRSF19	3.23E-05	-1.6823	1.6823
MARC2	4.93E-07	-1.6854	1.6854
LRP1B	1.17E-07	-1.6866	1.6866
RNF133	2.22E-09	-1.6877	1.6877
RGS2	4.25E-04	-1.6893	1.6893
SAMD13	1.60E-05	-1.6898	1.6898
FAM174B	5.09E-08	-1.6933	1.6933
GSTM5	2.75E-05	-1.6941	1.6941
C1orf115	2.41E-09	-1.6996	1.6996
GIPC2	9.91E-12	-1.7006	1.7006
HPGD	1.78E-05	-1.7008	1.7008
KIAA1324	2.13E-04	-1.7016	1.7016
UCP2	4.94E-05	-1.7028	1.7028
ACAT1	2.54E-08	-1.7060	1.7060
GCNT3	7.30E-05	-1.7063	1.7063
ABCA10	5.17E-05	-1.7167	1.7167
ACVR1C	2.19E-06	-1.7190	1.7190
SLCO4C1	2.19E-04	-1.7207	1.7207
PHYHD1	2.26E-06	-1.7218	1.7218
SERPINI1	4.92E-09	-1.7222	1.7222
PLEKHH1	1.67E-05	-1.7270	1.7270
CPT1A	1.60E-08	-1.7361	1.7361
CLIC6	6.02E-07	-1.7362	1.7362
TIMP4	4.14E-04	-1.7396	1.7396
KIAA1024	1.18E-08	-1.7415	1.7415
TMC5	2.61E-07	-1.7416	1.7416
TM7SF2	3.66E-11	-1.7432	1.7432
TSPAN1	1.06E-04	-1.7468	1.7468
DPYD	1.68E-07	-1.7489	1.7489
MKNK2	1.79E-09	-1.7512	1.7512
DACH1	1.53E-07	-1.7530	1.7530

CCDC110	1.08E-05	-1.7562	1.7562
PHLDA1	1.26E-05	-1.7564	1.7564
SERPINB1	5.49E-08	-1.7565	1.7565
TPK1	1.76E-10	-1.7596	1.7596
GALNT16	2.54E-06	-1.7788	1.7788
OAS2	8.11E-07	-1.7875	1.7875
NCALD	2.58E-04	-1.7891	1.7891
PDGFRB	1.72E-04	-1.7903	1.7903
C5orf27	7.65E-07	-1.7927	1.7927
GLRB	1.77E-06	-1.7943	1.7943
CEP41	1.93E-07	-1.7952	1.7952
FUT2	6.48E-06	-1.8036	1.8036
FHL1	1.32E-04	-1.8058	1.8058
TCEA3	4.18E-08	-1.8072	1.8072
INPP5D	7.58E-05	-1.8085	1.8085
APOD	4.68E-04	-1.8099	1.8099
PLLP	4.33E-06	-1.8108	1.8108
KLRF1	1.67E-04	-1.8170	1.8170
SLCO2A1	3.92E-07	-1.8183	1.8183
KLK11	3.45E-05	-1.8236	1.8236
CPD	2.15E-10	-1.8248	1.8248
MCFD2	9.24E-13	-1.8335	1.8335
SLC19A3	1.29E-04	-1.8337	1.8337
SLC31A1	1.24E-09	-1.8361	1.8361
GALNT5	2.15E-04	-1.8386	1.8386
GALNT6	1.42E-09	-1.8389	1.8389
FGF12	1.36E-07	-1.8413	1.8413
TLR3	2.46E-09	-1.8420	1.8420
DUSP1	4.29E-04	-1.8482	1.8482
EPB41L4A	9.47E-08	-1.8487	1.8487
CFD	2.86E-06	-1.8539	1.8539
GATA3	3.22E-06	-1.8607	1.8607
ADHFE1	1.97E-08	-1.8620	1.8620
PLTP	6.50E-07	-1.8647	1.8647
SPATS2L	1.07E-07	-1.8685	1.8685
ESRRG	6.77E-07	-1.8781	1.8781
PART1	7.30E-05	-1.8789	1.8789
FAM107A	9.35E-09	-1.8802	1.8802
ATP8B1	6.96E-09	-1.8823	1.8823
GLRX	1.68E-06	-1.8853	1.8853
PPARG	5.19E-05	-1.8872	1.8872
IL33	2.56E-06	-1.8879	1.8879
FURIN	8.27E-09	-1.8999	1.8999

ABCA6	4.54E-04	-1.9011	1.9011
TFAP2B	5.70E-08	-1.9104	1.9104
G0S2	8.62E-07	-1.9135	1.9135
ATP2A3	1.96E-06	-1.9136	1.9136
CIDEC	5.51E-05	-1.9161	1.9161
ATP1B1	6.54E-05	-1.9188	1.9188
FXYD2	1.67E-09	-1.9241	1.9241
ARHGAP24	9.06E-08	-1.9242	1.9242
GATM	2.18E-06	-1.9250	1.9250
PLAG1	9.85E-04	-1.9289	1.9289
TMEM45B	1.57E-06	-1.9384	1.9384
FABP3	3.40E-07	-1.9393	1.9393
LRRTM1	4.26E-09	-1.9427	1.9427
GPR15	2.28E-04	-1.9478	1.9478
FNDC5	1.21E-09	-1.9492	1.9492
LAMA4	1.31E-05	-1.9579	1.9579
SMCO4	1.14E-07	-1.9582	1.9582
ACACB	1.67E-07	-1.9587	1.9587
LRP2	5.63E-06	-1.9601	1.9601
SLC16A14	6.34E-09	-1.9666	1.9666
PLA2R1	3.55E-07	-1.9735	1.9735
CA12	2.53E-07	-1.9736	1.9736
HID1	5.39E-07	-1.9738	1.9738
IGF1	3.07E-06	-1.9835	1.9835
PPP1R1B	4.35E-08	-1.9846	1.9846
ARHGEF38	4.03E-05	-1.9925	1.9925
FIGN	3.19E-06	-2.0197	2.0197
CHST9	5.37E-04	-2.0257	2.0257
PHLDB2	2.58E-07	-2.0275	2.0275
PRKCH	1.26E-07	-2.0291	2.0291
CACNB2	4.25E-07	-2.0293	2.0293
GPR160	1.17E-06	-2.0294	2.0294
SLC20A2	4.49E-09	-2.0408	2.0408
CDH19	8.24E-08	-2.0451	2.0451
PDZK1IP1	9.93E-09	-2.0632	2.0632
SLC7A2	3.92E-06	-2.0644	2.0644
HSPB8	2.40E-04	-2.0667	2.0667
RNF128	6.44E-10	-2.0698	2.0698
ALDH2	4.00E-05	-2.0722	2.0722
MGLL	2.32E-10	-2.0735	2.0735
GALM	2.71E-07	-2.0744	2.0744
RNF150	5.88E-09	-2.0915	2.0915
RUNX1T1	5.59E-07	-2.0932	2.0932

ANGPT1	1.18E-08	-2.1055	2.1055
PLIN4	2.01E-05	-2.1083	2.1083
HDAC9	3.44E-07	-2.1085	2.1085
XBP1	8.74E-10	-2.1122	2.1122
WWC1	3.91E-08	-2.1174	2.1174
ADRA1A	1.39E-11	-2.1199	2.1199
ATP6V1C2	1.26E-04	-2.1206	2.1206
SNTB1	1.79E-09	-2.1258	2.1258
CHRDL1	3.82E-05	-2.1282	2.1282
PLIN1	1.77E-05	-2.1320	2.1320
CCL28	1.59E-05	-2.1321	2.1321
CRYBG3	5.80E-09	-2.1325	2.1325
PLEKHS1	9.27E-05	-2.1344	2.1344
MME	4.66E-05	-2.1345	2.1345
CXCL17	7.45E-07	-2.1398	2.1398
SLC41A2	1.93E-06	-2.1557	2.1557
ADIRF	2.60E-10	-2.1745	2.1745
MANSC1	6.41E-06	-2.1880	2.1880
ACSL1	2.41E-05	-2.1979	2.1979
TMPRSS11E	1.47E-04	-2.2072	2.2072
CKMT2	2.15E-07	-2.2077	2.2077
PKDCC	5.16E-10	-2.2116	2.2116
RGCC	1.22E-07	-2.2125	2.2125
STEAP4	1.03E-04	-2.2154	2.2154
PKHD1L1	2.93E-11	-2.2195	2.2195
PRKACB	1.09E-09	-2.2282	2.2282
SCD	5.14E-06	-2.2347	2.2347
FGF10	2.15E-10	-2.2416	2.2416
IGKC	7.58E-04	-2.2419	2.2419
PDE3A	1.90E-06	-2.2429	2.2429
ASPA	4.95E-07	-2.2538	2.2538
NXPE1	3.61E-09	-2.2619	2.2619
PTCH2	1.34E-06	-2.2727	2.2727
SPDEF	1.77E-05	-2.2899	2.2899
LRRK2	6.68E-09	-2.2990	2.2990
SLC9A1	4.20E-09	-2.3036	2.3036
FUT8	9.68E-07	-2.3038	2.3038
EYA1	4.05E-09	-2.3107	2.3107
AOC3	6.10E-06	-2.3301	2.3301
SCN7A	1.18E-06	-2.3345	2.3345
CSN3	2.24E-07	-2.3375	2.3375
ELL2	1.31E-09	-2.3381	2.3381
LRRC26	1.28E-09	-2.3489	2.3489

ACKR4	1.05E-06	-2.3549	2.3549
DPT	2.44E-04	-2.3604	2.3604
CCDC68	2.56E-12	-2.3689	2.3689
PTGS2	2.85E-05	-2.3753	2.3753
KCNJ16	5.59E-09	-2.3778	2.3778
DUSP6	8.88E-10	-2.3794	2.3794
CLU	3.32E-07	-2.3896	2.3896
TPD52L1	1.44E-08	-2.3939	2.3939
ADH1A	2.63E-07	-2.3941	2.3941
EGF	5.58E-04	-2.4094	2.4094
ATP2B2	8.24E-10	-2.4201	2.4201
ABI3BP	3.28E-04	-2.4278	2.4278
RAP1GAP	2.11E-08	-2.4397	2.4397
PLCB4	1.81E-05	-2.4409	2.4409
FAM46C	3.33E-06	-2.4434	2.4434
C1orf168	2.22E-09	-2.4438	2.4438
CSN1S2AP	5.68E-10	-2.4473	2.4473
BLM	3.84E-08	-2.4502	2.4502
SOAT1	8.25E-10	-2.4506	2.4506
CTSC	4.88E-06	-2.4595	2.4595
HSD11B2	3.51E-07	-2.4606	2.4606
DHRS7	1.10E-10	-2.4852	2.4852
SLC39A8	3.05E-09	-2.4874	2.4874
ALDH1L2	2.39E-06	-2.5005	2.5005
CSN1S1	9.26E-07	-2.5030	2.5030
MARC1	7.94E-12	-2.5080	2.5080
GNE	1.23E-07	-2.5092	2.5092
ARFGEF3	2.97E-06	-2.5159	2.5159
POF1B	2.12E-08	-2.5193	2.5193
ATP6V0A4	4.72E-06	-2.5237	2.5237
SEC11C	4.03E-11	-2.5292	2.5292
C14orf39	8.31E-07	-2.5400	2.5400
LCN2	1.10E-08	-2.5405	2.5405
MYOC	2.41E-09	-2.5442	2.5442
ENPP3	4.32E-07	-2.5463	2.5463
AGFG2	2.92E-09	-2.5513	2.5513
ABCA9	3.11E-06	-2.5528	2.5528
MLPH	3.01E-09	-2.5715	2.5715
PALMD	1.81E-08	-2.5741	2.5741
ALDH1L1	1.66E-10	-2.5828	2.5828
MYRIP	4.10E-12	-2.5879	2.5879
MSMB	1.41E-08	-2.5903	2.5903
NUCB2	1.10E-08	-2.5929	2.5929

ABCD2	3.91E-08	-2.5973	2.5973
GNA14	4.48E-09	-2.5985	2.5985
CNTN5	1.35E-04	-2.6034	2.6034
LTF	2.17E-04	-2.6133	2.6133
SLC13A5	2.86E-04	-2.6203	2.6203
AMACR	4.95E-08	-2.6212	2.6212
CHI3L2	1.03E-08	-2.6223	2.6223
C9orf152	5.97E-07	-2.6403	2.6403
MAOA	1.07E-08	-2.6688	2.6688
FOXP2	7.53E-09	-2.6803	2.6803
HBA1	2.66E-04	-2.6904	2.6904
ANO5	7.38E-08	-2.6964	2.6964
TNFRSF11A	1.50E-11	-2.6990	2.6990
PIK3AP1	1.11E-09	-2.7136	2.7136
MAN1A1	1.67E-07	-2.7145	2.7145
PDE8B	3.15E-10	-2.7588	2.7588
BHLHA15	4.04E-11	-2.7646	2.7646
LIFR	6.36E-08	-2.7681	2.7681
RNASE8	8.31E-08	-2.7828	2.7828
WDR72	4.87E-05	-2.7906	2.7906
ADGRG2	8.47E-06	-2.8019	2.8019
HGD	3.28E-07	-2.8122	2.8122
RRAGD	4.32E-07	-2.8133	2.8133
PDK4	3.91E-06	-2.8333	2.8333
FBP1	3.59E-12	-2.8539	2.8539
SIDT1	2.36E-09	-2.8736	2.8736
IQGAP2	4.71E-08	-2.8737	2.8737
KCNJ15	6.53E-07	-2.8914	2.8914
VPS36	1.45E-11	-2.8928	2.8928
IGSF10	2.03E-12	-2.8945	2.8945
ACADL	9.12E-11	-2.8947	2.8947
RBP4	2.27E-06	-2.9009	2.9009
SLC1A2	8.55E-10	-2.9155	2.9155
ACPP	1.24E-08	-2.9212	2.9212
ERBB4	3.05E-06	-2.9213	2.9213
PARM1	3.61E-09	-2.9269	2.9269
CRACR2A	2.43E-07	-2.9439	2.9439
FAM3D	8.04E-11	-2.9456	2.9456
ACSS3	2.55E-07	-2.9491	2.9491
KIAA0040	2.34E-08	-2.9497	2.9497
TBC1D30	3.59E-12	-2.9532	2.9532
ABCA8	1.25E-05	-2.9533	2.9533
SLPI	7.32E-09	-2.9621	2.9621

KAT2B	1.42E-09	-2.9906	2.9906
C8orf4	1.62E-12	-3.0034	3.0034
THRSP	2.23E-08	-3.0214	3.0214
GPD1	1.82E-08	-3.0567	3.0567
TRPV6	1.72E-09	-3.0697	3.0697
C1QTNF3	2.44E-06	-3.0724	3.0724
HBB	3.47E-04	-3.0764	3.0764
AQP3	4.41E-06	-3.0834	3.0834
BPIFB2	1.18E-06	-3.0838	3.0838
SH3BGRL2	1.26E-08	-3.1101	3.1101
CADPS2	4.03E-11	-3.1212	3.1212
FAM171B	1.48E-09	-3.1216	3.1216
PAX9	1.79E-09	-3.1622	3.1622
FAM20A	8.73E-11	-3.2341	3.2341
PON3	1.90E-08	-3.2947	3.2947
LMOD3	7.53E-10	-3.2957	3.2957
NXPE2	2.35E-08	-3.3227	3.3227
MUC15	5.98E-06	-3.3325	3.3325
SIX1	2.60E-10	-3.3408	3.3408
SLC31A2	7.69E-11	-3.3604	3.3604
FAM129A	6.88E-09	-3.3859	3.3859
DEPTOR	3.89E-09	-3.3982	3.3982
DEFB1	1.49E-08	-3.4425	3.4425
ASB5	1.10E-10	-3.5088	3.5088
PDE3B	6.78E-09	-3.5093	3.5093
TSPAN8	7.02E-06	-3.5492	3.5492
OR5G5P	2.35E-09	-3.5592	3.5592
PEBP4	1.45E-11	-3.5858	3.5858
SLC5A5	3.28E-10	-3.6691	3.6691
SLC26A9	4.53E-09	-3.6694	3.6694
BPIFB1	5.53E-07	-3.6938	3.6938
WNT5A	5.23E-08	-3.7165	3.7165
ETNPPL	3.53E-10	-3.7292	3.7292
ALDH1A1	4.12E-09	-3.7359	3.7359
ADH1C	3.27E-10	-3.7416	3.7416
AQP5	4.07E-08	-3.7511	3.7511
ETV1	5.31E-08	-3.7599	3.7599
NOSTRIN	1.70E-09	-3.7703	3.7703
PRH1	7.12E-08	-3.8026	3.8026
DNER	3.64E-10	-3.8163	3.8163
PRR4	1.52E-11	-3.8213	3.8213
MUCL1	4.26E-04	-3.8383	3.8383
TESC	2.77E-12	-3.8422	3.8422

CD36	4.15E-06	-3.8553	3.8553
LYZ	4.55E-06	-3.9082	3.9082
FDCSP	1.84E-05	-3.9341	3.9341
MEIS2	1.98E-08	-3.9697	3.9697
SLC37A2	3.46E-13	-3.9783	3.9783
PRUNE2	2.36E-12	-3.9792	3.9792
CRISP3	2.65E-05	-4.0175	4.0175
JCHAIN	6.94E-04	-4.0685	4.0685
MGAM2	2.60E-08	-4.1484	4.1484
ANGPTL1	4.10E-12	-4.1550	4.1550
MAOB	1.58E-11	-4.1875	4.1875
WIF1	2.54E-10	-4.2623	4.2623
ADH1B	1.48E-09	-4.3561	4.3561
FAM3B	6.08E-09	-4.4065	4.4065
FMO6P	1.25E-09	-4.4412	4.4412
CST2	1.59E-06	-4.4693	4.4693
DNASE2B	3.33E-11	-4.4696	4.4696
ADIPOQ	7.10E-10	-4.5926	4.5926
KLK1	1.38E-10	-4.6604	4.6604
SMR3A	3.15E-08	-4.6900	4.6900
SLC22A3	6.92E-13	-4.6923	4.6923
ODAM	1.89E-08	-4.6999	4.6999
TCN1	8.28E-09	-4.7732	4.7732
ZG16B	1.32E-07	-4.8121	4.8121
CST5	6.80E-08	-4.8871	4.8871
AMY1A	3.42E-08	-4.9408	4.9408
PRB4	6.61E-08	-5.1840	5.1840
NXPE4	9.24E-13	-5.3493	5.3493
CA6	2.69E-06	-5.3520	5.3520
PRB3	1.48E-07	-5.4192	5.4192
PIGR	3.47E-09	-5.4739	5.4739
PRB1	4.85E-07	-5.4986	5.4986
DMBT1	2.03E-12	-5.5563	5.5563
MUC7	3.17E-06	-5.5847	5.5847
FABP4	5.59E-07	-5.5902	5.5902
PRR27	9.34E-08	-5.7596	5.7596
PIP	1.07E-08	-5.9544	5.9544
DPP4	4.34E-11	-6.1073	6.1073
SMR3B	9.21E-05	-6.1581	6.1581
LPO	1.45E-11	-6.2040	6.2040
PRH2	2.87E-07	-6.4871	6.4871
HTN3	1.50E-04	-6.8118	6.8118
HTN1	6.10E-05	-6.8163	6.8163

BPIFA2	1.14E-08	-6.9060	6.9060

ACC, adenoid cystic carcinoma; FDR, false discovery rate; FC, fold change.
